# Metamaterials and Metasurfaces for Sensor Applications

**DOI:** 10.3390/s17081726

**Published:** 2017-07-27

**Authors:** Yohan Lee, Sun-Je Kim, Hyeonsoo Park, Byoungho Lee

**Affiliations:** Inter-University Semiconductor Research Center and School of Electrical and Computer Engineering, Seoul National University, Gwanak-Gu Gwanakro 1, Seoul 08826, Korea; brahms0827@gmail.com (Y.L.); donut9080@gmail.com (S.-J.K.); psoohyeon@gmail.com (H.P.)

**Keywords:** metamaterial, metasurface, refractive index sensing, biosensor, light analysis, polarimetry, spectroscopy

## Abstract

Electromagnetic metamaterials (MMs) and metasurfaces (MSs) are artificial media and surfaces with subwavelength separations of meta-atoms designed for anomalous manipulations of light properties. Owing to large scattering cross-sections of metallic/dielectric meta-atoms, it is possible to not only localize strong electromagnetic fields in deep subwavelength volume but also decompose and analyze incident light signal with ultracompact setup using MMs and MSs. Hence, by probing resonant spectral responses from extremely boosted interactions between analyte layer and optical MMs or MSs, sensing the variation of refractive index has been a popular and practical application in the field of photonics. Moreover, decomposing and analyzing incident light signal can be easily achieved with anisotropic MSs, which can scatter light to different directions according to its polarization or wavelength. In this paper, we present recent advances and potential applications of optical MMs and MSs for refractive index sensing and sensing light properties, which can be easily integrated with various electronic devices. The characteristics and performances of devices are summarized and compared qualitatively with suggestions of design guidelines.

## 1. Introduction

Electromagnetic (EM) metamaterials (MMs) and metasurfaces (MSs) are rising research fields nowadays owing to their anomalous and largely tunable properties of light scattering in ultracompact volume. Performances of MMs and MSs are dependent on their subwavelength separation and geometric parameters of each meta-atom (MA), a fundamental constituent of MM and MS [1,2,3,4]. MAs are composed of one or more subwavelength nanostructures with noble metals or high index dielectrics. They are engineered to exhibit desired effective local optical responses, which can be expressed in terms of amplitude and phase or electric and magnetic polarizabilities [1,3]. Exotic functionalities such as negative refractive index (RI) [5,6,7,8,9,10,11], nearly perfect absorption [12,13,14,15,16,17,18,19,20,21,22], and transmission [23,24,25,26,27,28] have been demonstrated with various potential applications. Among various potential applications including superlens [29,30,31], slow light [32,33,34], and cloaking devices [35,36,37,38,39,40], RI bio-sensing is the most realistic and representative application of them. Bio-molecular interactions that occur in analyte layers result in changes of RIs. The EM RI sensor offers distinguishing opportunities for sensitive and label-free biochemical assays so that it can play a crucial role in various chemical and biological sensing technologies.

By engineering individual MAs and their arrangements, resonant EM spectra can be largely tuned, which are dominated by surrounding environment. Owing to such resonant properties, RI of surrounding biomolecular analyte can be probed with variation of scattered output spectra. Hence, it is essential to design appropriate sensitive MAs at the target wavelengths and certain configurations. Moreover, MM- and MS-based RI sensing platforms have several advantages over conventional SPP-based biosensors. At first, as RI variation is detected by macroscopic optical responses, mainly reflection or transmission of focused input beams, MM- and MS-based RI sensors are better than SPP-based ones with respect to fabrication tolerance and stability of readout signal [41,42,43]. Secondly, periodic arrangements of MAs enable lower radiative damping and higher quality factor with interesting physical mechanisms including plasmonically induced transparency [23,24,25], and Fano resonances [44,45]. Lastly, it would be possible to expand functionalities of single nanophotonic RI sensor with MM or MS. Delicate and elaborate compositions of multiple different MAs in a unitcell or supercell can yield multiple resonances and broadband slow light phenomena which are hard to be allowed in SPP-based sensors [32,46,47].

On the other hand, sensing light itself using light matter interactions has also been a thoroughly studied topic in optics society. Based on interference between multiple beams, anisotropic scattering, and dispersive responses from certain EM devices, fundamental properties of arbitrary incident EM wave can be sensed and analyzed. Polarization and spectral composition are main characteristics of interest and there are commercial bulk-optic devices to analyze them, which are called polarimeter and spectrometer. As demands on integration and miniaturization of such light sensing components for electronic devices have been explosively increasing, MMs and MSs have been expected to be a promising platform to implement those functions with compact volume and lightweight [48,49,50,51].

In this review, we introduce recent advances and potential applications of EM MM and MS for various sensors. We mainly classify sensor applications into two major objects of MMs and MSs sensing: RI sensing with optical response and sensing properties of light itself. At first, RI sensing methods are discussed with respect to their physical mechanisms and sensing capabilities. Surface-enhanced Raman scattering (SERS), Fano resonance (FR), and hyperbolic metamaterials (HMM) are presented and their applications for RI sensing are proposed. Performances of those configurations are compared and potential novel methods of RI sensing based on holographic phase gradient (HPG) are discussed. Secondly, various functionalities of MSs as ultrathin flat light analyzer are discussed. Analysis on light properties such as polarization state and spectrum using MSs are explained.

## 2. Refractive Index Sensing

### 2.1. Sensors Based on Surface-Enhanced Raman Scattering

**Figure 1:**
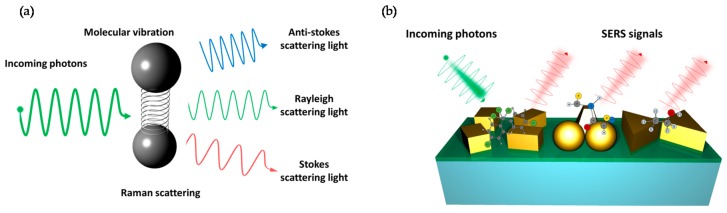
(**a**) The principle of Raman scattering; (**b**) Illustration of SERS sensing. Roughened metal surface or metallic nanostructures can enhance Raman signals and they can be applied to biological and chemical sensing.

Raman scattering is the inelastic scattering of photons by molecules which are excited to higher vibrational or rotational energy levels (See Figure 1a). The spectra of the Raman-scattered light have been widely used for material identification and analysis because it depends on the molecular states. Especially, it has an evident merit that can be used to detect and analyze not only simple materials, including solids, liquids, and gases, but also highly complex materials such as human tissue, biological organisms, and chemical materials. Thanks to the invention and development of lasers from 1960s, Raman spectroscopy has become an important measurement method to provide a structural fingerprint by which molecules can be identified in various chemical processes. 

Despite its versatileness, it has taken a long time to apply Raman scattering directly to the sensor due to its inherently small scattering cross-section. When scattering occurs between matters and light, most photons are elastically scattered, called Rayleigh scattering, such that the scattered photons have the same frequency as the incident photons. Only a small fraction of the scattered photons, approximately 1 in 10 million, are scattered by an excitation with the scattered photons having a frequency different from that of incident photons. Therefore, due to the low probability of Raman scattering of molecules, satisfactory Raman spectra are hardly obtained in low sample concentration environments, such as in thin films coated with dilute solutions or monolayers. Another problem is that samples exhibiting color or fluorescence cannot be easily investigated with Raman spectra, unlike pure or colorless samples. Also, it is so difficult to obtain accurate outcomes in case of a sample which generates background-fluorescent signals when a laser in a visible region is illuminated if they are stronger than Raman scattering signals of the sample itself. In general, non-resonant Raman scattering cross-sections are typically about 10^−30^ cm^2^/molecule and 14–15 orders of magnitude lower than that of fluorescence [52]. For these reasons, Raman spectroscopy has been used only in special fields and has not been popular as a typical analysis method until 1960s. In 1974, Fleischmann et al. observed that Raman signals of pyridine absorbed on electrochemically roughened silver were enhanced [53]. A series of experiments in 1977 [54,55] confirmed that Raman signals can be greatly increased when molecules are very close to rough metal surfaces or nanostructures. It was called surface-enhanced Raman scattering (SERS) or surface-enhanced Raman spectroscopy (See Figure 1b).

For a few decades, to obtain more amplified signals, researchers have been basically using noble metal such as gold or silver, and various nanostructures as plasmonic antennas. This is because metal nanoantennas have an ability to support localized surface plasmons (LSPs), i.e., non-propagating excitations of the conduction electrons coupled to the EM field [56]. From an EM point of view, owing to the small Stokes shift, one usually assumes that the radiative Stokes field is same as enhancement of the exciting field, thus the Raman cross section (*R*) can be expressed by $R={\left|E_{loc}\right|}^{4}/{\left|E_{i}\right|}^{4}$, where $\left|E_{loc}\right|$ is the local field amplitude of the Raman active site and $\left|E_{i}\right|$ is the field of incidence [57,58]. Researchers have introduced the concept of enhancement factor (EF), which is one of the most important figures for characterizing the SERS effect. 

It is well known that in the close vicinity of the metal surface, the EM field enhancement by LSPs is especially strong at sharp tips [59,60,61,62], nanogaps such as aperture or pores [63,64,65,66,67], and inter-particles [68,69,70,71]. Researchers have focused their extensive efforts on SERS substrates design such as individual size, shape, periodicity, and spacing between layers, with strongly localized EM field which can excite strong Raman scattering [41,42,72]. Thanks to their dedications, strong SERS with EF of 10^8^–10^14^ times has been observed, which allows to detect single molecules. The following table summarizes the features of various nanostructures for SERS (See Table 1).

**Figure 2:**
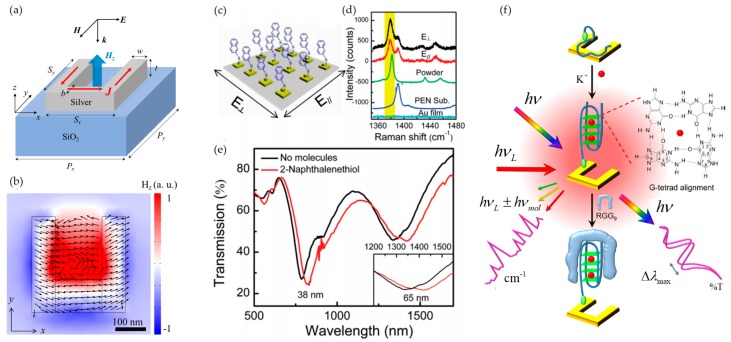
(**a**) A schematic illustration of SRR. Structural parameters are described. Magnetic field is generated along *z*-axis when *x*-polarized incidence is coming along *z*-axis; (**b**) *H_z_*-field profile on resonance. It shows the generation of current loops (black arrows). The resonance conditions are as follow: *λ* = 1300 nm, *S_x_* = *S_y_* = 320 nm, *P_x_* = 504 nm, *P_y_* = 480 nm, *w* = 80nm, *t* = 30 nm, *b* = 128 nm; (**c**) A schematic illustration of chemical sensor of 2-naphtalenethiol using MM; (**d**) Raman spectra of 2-naphtalenethiol on various substrate environments; (**e**) Transmission spectra with and without 2-naphtalenethiol. The inset is the zoom-in view of the spectra around the magnetic resonance mode. (**f**) Schematic configuration of vis-NIR SRR MMs for detection and identification of biomolecules. This SRR sensor was designed to functionalize with AS1411, an effective anticancer drug. Parts (**c**–**e**) are reprinted with permission from Reference [84]. Copyright (2011) American Chemical Society. Part (**f**) is reprinted with permission from Reference [85]. Copyright (2013) American Chemical Society.

Despite the achievement of increasing the EF of SERS signal through various SERS substrate designs, its application is still restricted by poor reproducibility of the enhanced signals and structural inelasticity. Most SERS substrates can work for individual excitation wavelength only, thus the different types of substrates are needed for analysis by different excitation wavelengths. In terms of being able to design optical resonance characteristics, MMs and MSs can allow for multi-functionalities as SERS substrates. To understand it, we introduce the split ring resonator (SRR) structure, which is one of the most renowned structure as MA of MM [9,73,74,75,76,77,78,79,80,81,82]. The structure of which shape is like “C” (See Figure 2a) has been of interest to many researchers since early 2000 because it allows for the implementation of substances with optical properties that do not occur in nature. There are a series of studies on implementation of negative permittivity and permeability from gigahertz to vis-to-near-infrared (vis-NIR) frequencies [9,75,76,77,78,79,80,81,82]. The principle of SRR is as follows: When the incidence passes the SRR, a loop current is formed along the structure. Excited circular loop of current operates as a magnetic dipole and each ring has an individual tailored response controlled by gap. It was confirmed that a current loop and magnetic field are generated when *x*-polarized incidence is coming as shown in Figure 2b. However, EM wave interacts as if homogeneous medium when many SRRs are arranged with periodicity of subwavelength scale, thus one can control the magnetic response over that found in nature at lager scale. Besides the SRR, various types of nanostructures, which are designed to obtain the desired optical responses, have been actively suggested. 

MMs and MSs are good candidate to be used for SERS substrate because metal surface of them can excite surface plasmon polariton (SPP), which help to strengthen Raman signals, and EM responses in the whole range of vis-NIR spectra regime to detect biomolecules and protein can be controlled by engineering MAs [83]. Xu et al. designed SERS substrate by SRR-based MM and achieved chemical sensing [84]. Cao et al. demonstrated a tunable vis-NIR MMs-based nanosensor with SERS molecular detection [85]. SRR was designed to have two resonance peaks, electric and magnetic resonances, respectively and the proposed sensor conducted two parallel acquisitions of optical transmission and SERS spectra at the bio-interface. The EF was found to be about 6.5 × 10^7^, which is strong enough to allow a sensitivity of single molecule detection. In addition, they achieved for the first time the use of MMs as SERS-based logic gate operation, serving as sensors of mercury ions with high sensitivity, by using SRR structure [86]. 

**Figure 3:**
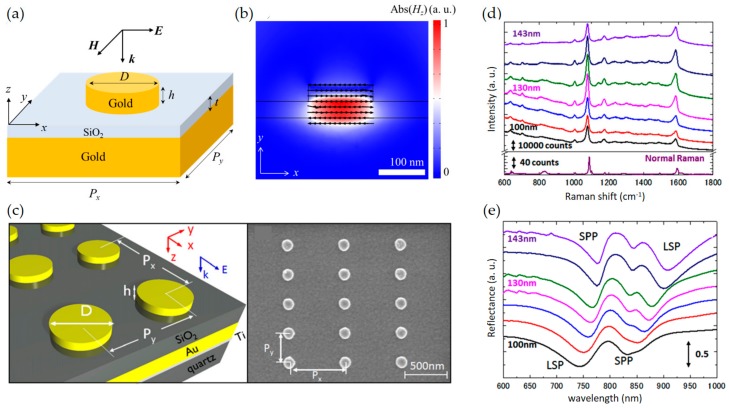
(**a**) Schematic configuration of nanodisk-based MM; Structural parameters are described. (**b**) Absolute value of *H_z_*-field profile on resonance. It shows the generation of current loops (black arrows). The resonance conditions are as follow: *λ* = 879 nm, *D* = 130 nm, *h* = 30 nm, *t* = 30nm, *P_x_* = 580 nm, *P_y_* = 300 nm; (**c**) Schematic illustration of double-resonances gold nanodisk array substrate (left panel) and its SEM image (right panel); (**d**) SERS spectra on double-resonances. The diameter changes from 100 to 143 nm (black to purple); (**e**) Reflectance spectra corresponding to (**d**). Parts (**c**–**e**) are reprinted from Reference [87], with the permission of AIP Publishing.

Also, among various types of MM, it is worthy to note that nanodisks have been used for SERS substrate [87,88]. This type of nanostructure has several advantages such as easy fabrication, polarization-independent property, and relatively simple control of parameters (its radius and thickness) (see Figure 3a). In addition, MMs can control the resonance wavelength of the SPP and the LSP by controlling array period and diameter of particles [87]. It has been suggested that localized surface plasmon resonance (LSPR) wavelength should be located between Raman scattering wavelength and the excitation wavelength [89] because the SERS EF is proportional to the product of field intensity at both Raman scattering and excitation wavelengths (See Figure 3b). Y. Chu et al. suggested double-resonance plasmon substrates consisting of a gold disk array, a dielectric spacer, and a gold film and obtained the EF of 7.2 × 10^7^, which is more than two orders of magnitude larger than that measured on a gold nanoparticle array on a simple glass substrate [90]. In addition, nanoporous plasmonic MMs have attracted interests due to its easy fabrication such as dealloying and templating. Jiao et al. achieved the EF of 5.0 × 10^7^ by using two dimensional patterned nanoporous gold film [91] and Zhang et al. demonstrated that nanoporous MMs-based sensor shows higher EF than that of mesopore or nanohole [92]. 

### 2.2. Sensors Based on Fano Resonances

**Figure 4:**
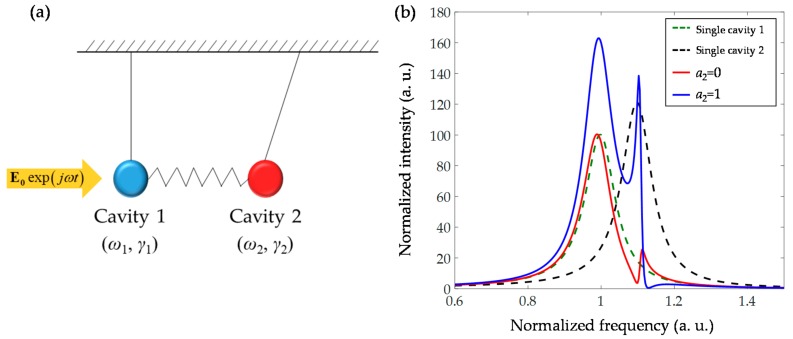
(**a**) Schematic illustration of classical coupled oscillators. Two oscillators are linked with harmonic potential; (**b**) Real values of *x*_2_ in Equation (2) are plotted. Each curve refers to cases when *a*_2_ = 0 (red line) and *a*_2_ = 1 (blue line), respectively. To compare with single oscillator system, Lorentzian line-shapes of single cavity 1 (green dashed line) and 2 (black dashed line) with Q-factor of 10 are plotted, too (*ω*_1_ = 1, *ω_2_* = 1.1, *γ*_1_ = 0.1, *γ*_2_ = 0.01, *κ* = 0.25).

Fano resonances are defined as a type of resonant scattering phenomenon that gives rise to an asymmetric line-shape [93]. This asymmetric line-shape is usually attributed by interference between two scattering amplitudes, one due to scattering within a continuum of states and the second due to an excitation of a discrete state, which means the resonant process [94,95]. We present simple coupled oscillators to explain Fano resonance briefly as shown in Figure 4a. Each oscillator acts as cavity and has natural frequencies *ω*_1_ and *ω*_2_, corresponding to the cavity 1 and 2, respectively. The equations of motions are solved in terms of displacements *x*_1_ and *x*_2_ from the equilibrium positions:

(1) $${\ddot{x}}_{1}+\gamma_{1}{\dot{x}}_{1}+\omega_{1}{}^{2}x_{1}-\kappa^{2}x_{2}=a_{1}e^{-j\omega t},\;{\ddot{x}}_{2}+\gamma_{2}{\dot{x}}_{2}+\omega_{2}{}^{2}x_{2}-\kappa^{2}x_{1}=a_{2}e^{-j\omega t},$$

where *κ* describes the coupling coefficient of the oscillators, *γ*_1_ and *γ*_2_ are the damping coefficients. The solution of Equation (1) on steady-state is given by

(2) $$\begin{array}{l} x_{1}=\frac{a_{1}\left(\omega_{2}{}^{2}-\omega^{2}-j\gamma_{2}\omega \right)+a_{2}\kappa^{2}}{\left(\omega_{1}{}^{2}-\omega^{2}-j\gamma_{1}\omega \right)\left(\omega_{2}{}^{2}-\omega^{2}-j\gamma_{2}\omega \right)-\kappa^{4}}e^{j\omega t},\\ x_{2}=\frac{a_{2}\left(\omega_{1}{}^{2}-\omega^{2}-j\gamma_{1}\omega \right)+a_{1}\kappa^{2}}{\left(\omega_{1}{}^{2}-\omega^{2}-j\gamma_{1}\omega \right)\left(\omega_{2}{}^{2}-\omega^{2}-j\gamma_{2}\omega \right)-\kappa^{4}}e^{j\omega t}. \end{array}$$

In this classical harmonic model, Fano resonance usually occurs when incoming EM wave excites only broad mode *x*_1_, while the narrow resonance mode *x*_2_ is excited just owing to the coupling. This situation means that only one cavity is directly driven by EM wave where *a*_2_ is zero. Meanwhile, in case that EM wave excite both broad and narrow modes where *a*_2_ is not zero, the Fano line-shape also appears and even sharper. Figure 4b shows specifically the difference between each case.

**Figure 5:**
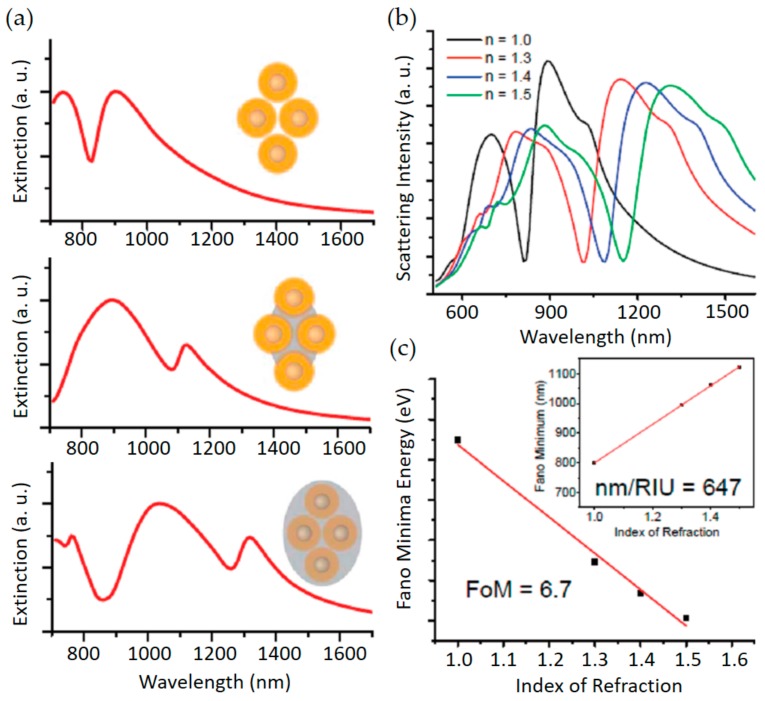
Calculated spectra of quadrumers in different dielectric environments. (**a**) The dip of Fano resonance is highly sensitive to the surrounding; (**b**) Scattering spectra of a quadrumer embedded in environments of different index of refraction; (**c**) The calculated figure of merit. The inset shows the calculated sensitivity to be 647 nm/RIU. Reprinted with permission from Reference [94]. Copyright (2010) American Chemical Society.

This resonant process was studied in atomic physics and quantum mechanics for the first time, but recently, it has been actively conducted in nanoparticles or plasmonic nanostructures [96,97,98,99,100,101,102,103,104,105,106,107,108], and MMs [24,25,44,45,84,109,110,111,112,113,114,115,116,117,118,119,120,121,122,123,124,125,126] due to its unique optical properties, such as strong field enhancement and extremely narrow asymmetric linewidth. Especially, ultrahigh quality factor is suitable for sensor applications because narrower linewidth allows a lower molecular concentration to be detected and higher sensitivity in surrounding medium to be obtainable. Example for sensor application of Fano resonance is presented in Figure 5. Despite there are a lot of various kinds of sensors to detect thickness of layer, temperature, chemical substances, the scope of this section is restricted to only RI sensors based on Fano resonances in MMs. The following contents introduces current sensors based on Fano resonances.

#### 2.2.1. Symmetry Breaking 

**Figure 6:**
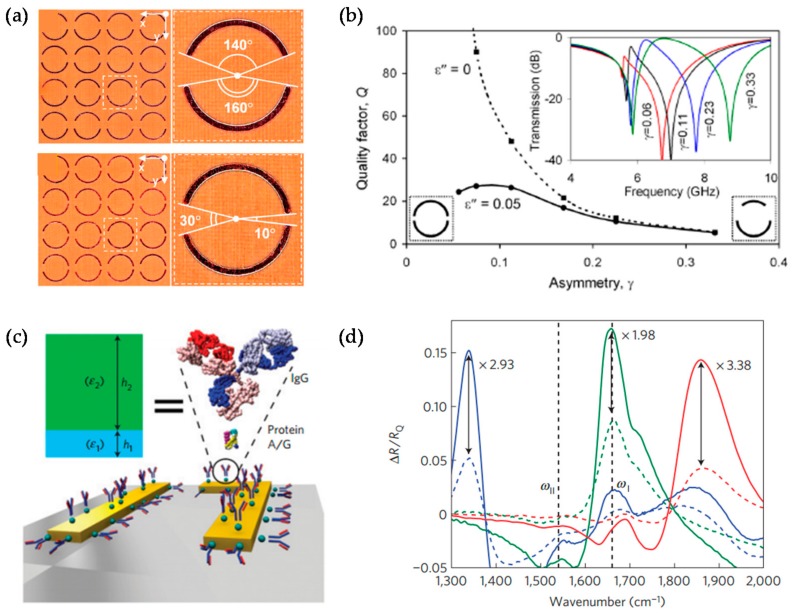
(**a**) Planar MMs with asymmetric split copper rings. The dashed boxes indicate unit-cell structure; (**b**) Quality factor of “trapped-mode” resonance as a function of the particle asymmetry *γ*, defined as the relative difference of the arcs’ lengths; (**c**) Schematic representations of proteins’ mono- and bilayers binding to the metal surface and the equivalent dielectric model; (**d**) Experimental spectra before (dashed lines) and after (solid lines) binding of IgG antibodies to three different Fano-resonant asymmetric MMs substrates immobilized by the protein A/G. Reprinted (**a**,**b**) with permission from Reference [115]. Copyright (2007) by the American Physics Society. Reprinted (**c**,**d**) by permission form Macmillan Publishers Ltd.: (Nature Materials) (Reference [123]), copyright (2012).

A general method to obtain easily Fano resonance in plasmonics is to break the symmetry of nanostructures [110,111,112,113,114,115]. For nanostructures that are small compared to the wavelength of incident light, dipole moments can be excited and its spectral profile by dipoles is usually broad. The first observation of Fano resonance in MMs was by N. I. Zheludev group in the microwave frequency range [115]. Asymmetric split rings (ASR) were used in unit-cell structure of MMs which act as resonator. By breaking symmetry, ‘narrow’ dark modes that are caused by higher-order oscillations can be excited and interact with the broad ‘bright’ modes. As shown in Figure 6b, the lifetime of dark mode depends on the degree of asymmetry *γ* and substrate loss tangent *ε*, and strong Fano resonance was obtained by changing two parameters. With similar structure, W. Zhang group obtained sharper Fano resonance peak in Terahertz frequency range [116,117], and achieved the level of 57,000 nm/RIU using thinner substrates [118]. Sensing in near-infrared frequency range was also achieved with ASR structure, which was improved to a slit type based on carbon nanotube MMs [119]. 

A number of other planar MMs by symmetry breaking have been recently reported [24,25,84,120,121,122,123,124]. At optical frequencies, N. Liu et al. experimentally demonstrated sharp reflectance peaks with EM-induced transparency (EIT), which is known as the elimination of absorption via quantum interference in an atomic medium [25]. Because it is known that EIT phenomenon can occur in classical system when optical resonators are coupled with one another [24,120], they proposed a design composed of asymmetrically arranged three nanoslits in a gold layer for easy fabrication. They confirmed that proposed sensor showed the sensitivity of 588 nm/RIU and figure of merit (FOM) of 5.3 introduced by Sherry et al. [121]. Y. Moritake et al. experimentally demonstrated sharp Fano resonance of which the highest quality factor is 7.34 by using asymmetric double-bars [122]. In case of infrared frequencies, C. Wu et al. designed infrared plasmonic MMs and suggested their use as biosensing platform [123]. As shown in Figure 6d, asymmetric *π*-shaped nanorod antenna was used and its structural parameters were determined for the control of the wavelength of the subradient resonance. They demonstrated biosensing and fingerprinting of target proteins by testing it on a well-defined ultrathin multiprotein layer. In terahertz frequency range, B. Reinhard et al. broke the symmetry by tilting the crosses and obtained a narrow lineshape [124]. 

#### 2.2.2. Hybrid Structures and Waveguide Gratings

Another approach is to design complex and hybrid structures for bright and dark modes hybridization. The fundamental principle of Fano resonance is a weak coupling and interference between bright and dark modes. In addition to breaking structural symmetry, Fano resonance is introduced by employing different materials [125,126,127,128,129,130]. In this case, the degree of asymmetry can be controlled by the materials’ properties like dielectric permittivities as well as geometrical parameters of structures. For example, Mock et al. presented an experimental analysis on plasmon coupling between a gold nanoparticles and gold film [125]. The effects of LSPR and optical spectral response was highly sensitive to the gap distance between the nanoparticles-film and showed the possibility of being applied as a sensor. In addition, for nanoshells on high dielectric permittivity substrates, the higher quadrupolar modes can induce a Fano resonance [126]. 

**Figure 7:**
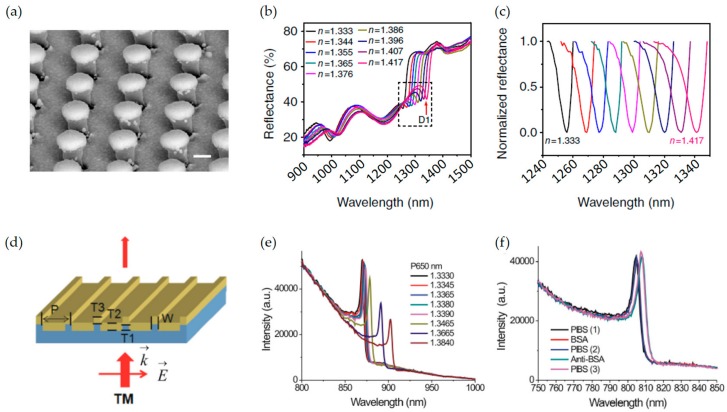
(**a**) Side-view SEM image of gold mushroom MMs. The scale bar is 200 nm; (**b**) Reflectance spectra of the gold mushroom array structure immersed in glycerine at the incidence angle of 33.3°; (**c**) Normalized relfectance spectra for D1 in the spectral region indicated with the dashed box in (**b**). The refractive index changes from 1.333 to 1.417 (black to pink); (**d**) A schematic configuration, depicting the geometrical parameters of the capped gold nanoslits, as well as the direction of the TM-polarized incidence with E and K vectors; (**e**) Intensity spectra of the capped gold nanoslit with a 650-nm period in carious water/glycerin mixtures for a normally-incident TM-polarized wave; (**f**) Measured intensity spectra for different surface conditions. Reprinted by permission from Macmillan Publishers Ltd.: (Nature Communications) Reference [128], copyright (2013).

In 2011, Zhang et al. theoretically analyzed the plasmon mode interactions of a silver nanocube on a glass substrate [127]. They showed that when the metallic nanocube is close to dielectric substrate, new plasmon mode is generated and a dark mode and a bright one are coupled in a nearly degenerate states. It showed the sensitivity of ~954 nm/RIU and high FOM of ~12–20, and these performances are enough to be applied to LSPR-based sensors. In 2013, Shen et al. suggested a design composed of gold mushroom-typed array for RI sensing [128] to overcome a low FOM value of LSPR-based sensor compared with propagating surface plasmon resonance (PSPR)-based sensor (See Figure 7a–c). Although the FOM value of LSPR-based sensors is generally 1–2 orders of magnitude smaller than those of PSPR-based sensor, the proposed structure showed a narrow FWHM of ~10 nm, FOM of ~108, and a high sensitivity of ~1010 nm/RIU. Meanwhile, dynamically reconfigurable sensor based on MMs was suggested by Amin et al. [129]. A hybrid graphene-gold Fano resonator which consists of a graphene patch and gold frame was used. Broad resonance by gold frame and narrow resonance by graphene were coupled and it introduced plasmonic EIT phenomenon and asymmetric Fano lineshape. Owing to graphene’s electrically tunable property, it could be applied to switching sensor through controlling an applied voltage.

In addition, using coupled waveguide grating structures can help to generate Fano resonance in MMs [130,131,132]. Because grating structure exhibits not only waveguide resonance (WR) but also Fabry-Pérot (FP) cavity resonance, many researchers have made high performance sensors by designing resonance peaks using it. Lee et al. reported Fano sensor composed of a single nanoslit with periodic grooves and it yields a FOM to 48 and the sensitivity of 615 nm/RIU [130]. In sequence, they experimentally demonstrated a new design composed of capped gold nanoslit array. Performances were also improved, the wavelength sensitivity is 926 nm/RIU and FOM value is up to 252 [131,132]. 

### 2.3. Sensors Based on Hyperbolic Metamaterials and Effective Medium Theory

**Figure 8:**
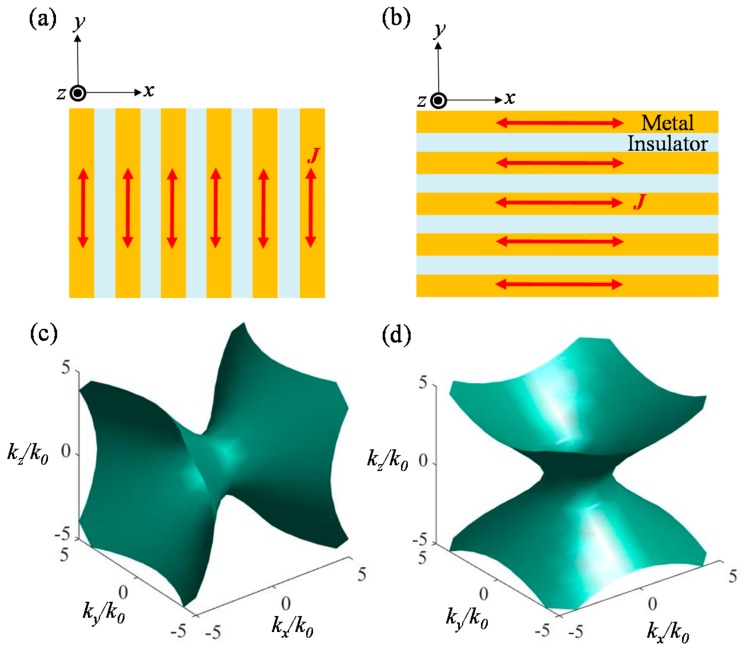
Schemes of basic HMM types whose optic axes are lying in: (**a**) horizontal (*x*-direction); and (**b**) perpendicular (*y*-direction) directions; (**c**,**d**) refer to isofrequency dispersion surfaces in the normalized momentum space for HMMs with (**a**,**b**) structures. Analytic expressions of plots in (**c**,**d**) are Equations (3) and (4).

HMMs are MMs which exhibit extraordinary anisotropy with hyperbolic dispersions of light. HMMs are formed by alternating arrangements of metals and insulators within subwavelength periods (Figure 8a–d) and represented with simple isofrequency dispersion curves as shown in Equations (3) and (4). Those simple dispersions are derived using effective permittivity tensors based on Maxwell Garnett effective medium theory (EMT) [133]. As shown in Figure 8 and Equations (3) and (4), there are two popular types of HMM structures depending on direction of negative effective permittivity. One exhibits negative vertical permittivity, while the other exhibits negative permittivity in horizontal direction. The direction of negative effective permittivity is determined by the allowed current direction (See the red arrows in Figure 8a,b), while filling factor and composition materials are properly selected for different signs of permittivities.

(3) $$1=\frac{k_{x}^{2}}{\varepsilon_{z}k_{0}^{2}}+\frac{k_{y}^{2}}{\varepsilon_{x}k_{0}^{2}}+\frac{k_{z}^{2}}{\varepsilon_{x}k_{0}^{2}}\;(\varepsilon_{x}>0,\;\varepsilon_{y}=\varepsilon_{z}<0),$$

(4) $$1=\frac{k_{x}^{2}}{\varepsilon_{z}k_{0}^{2}}+\frac{k_{y}^{2}}{\varepsilon_{z}k_{0}^{2}}+\frac{k_{z}^{2}}{\varepsilon_{x}k_{0}^{2}}\;(\varepsilon_{x}=\varepsilon_{y}<0,\;\varepsilon_{z}>0).$$

As diffractions with high effective indices in perpendicular direction are allowed in the two different HMMs, coupling between incident light and perpendicularly diffracting modes in HMMs would be extremely sensitive to external coupling environment and composition materials of HMMs [134].

**Figure 9:**
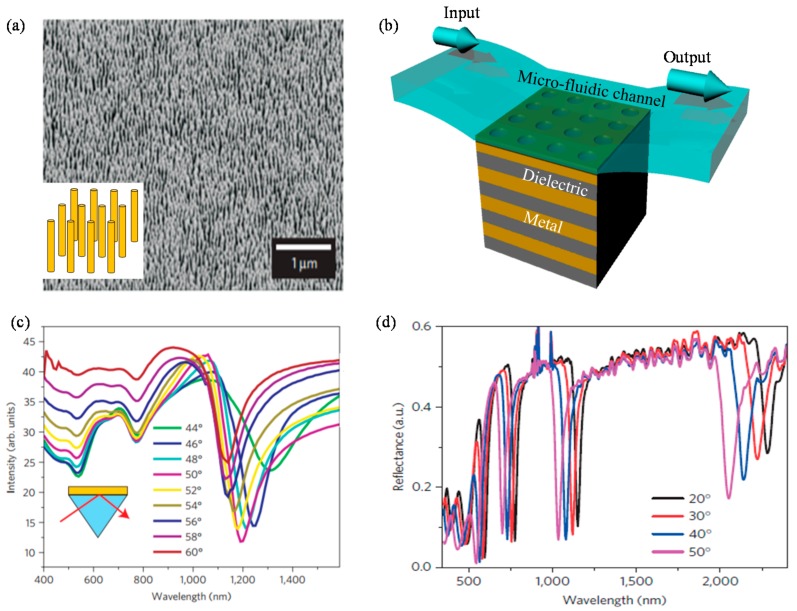
(**a**) Scanning electron microscopy image of standing gold nanorods assembly with subwavlength spacings made by A. V. Kabashin et al. [135]. The inset figure presents magnified scheme of standing gold nanorods; (**b**) Scheme of the grating coupled HMM consists of alternatively stratified metal nanolayers and dielectric nanolayers demonstrated by Sreekanth et al. [136]; (**c**) Attenuated total reflection (ATR) spectra for nine different incidence angles when nanorod HMM of (**a**) is located in water environment (*n* = 1.33); (**d**) ATR spectra of the grating coupled HMM depicted in (**b**) for four different incidence angles. The legends in (**c**,**d**) denote the incidence angles. Reprinted by permission from Macmillan Publishers Ltd.: (Nature Materials) (Reference [135]), copyright (2009) and (Nature Materials) (Reference [136]), copyright (2016).

Recently, there have been excellent achievements of applying the abovementioned sensitive characteristics of HMMs to RI sensors. In 2009, Kabashin et al. proposed standing nanorod assembly HMM (Figure 9a) as a label-free nanophotonic biosensor to enhance spectral sensitivity over 30,000 nm/RIU [125]. The proposed device operates in the ATR scheme where nanorod HMM is attached on the prism and covered with microfluidic channel while input broadband source is incident obliquely from the prism side. As plasmon-assisted guided mode propagates with highly confined electromagnetic field in the nanorod region, interaction between superstrate insulator layer (analyte layer) and the guided perpendicular mode is extremely enhanced. ATR spectra in Figure 9c shows that the guided mode resonance dips occur near the wavelength of 1200 nm by *p*-polarized light. Strongly coupled guided mode resonance and dependence of the effective guided mode index on the superstrate RI resulted in the great improvement in spectral sensitivity.

In 2016, Sreekanth et al. demonstrated grating coupled HMM device (Figure 9b) for both extremely high spectral sensitivity [136] and angular sensitivity [137] exceeding previous conventional surface plasmon resonance based RI sensors. They proposed the structure with HMM covered with a two-dimensional nanohole grating and microfluidic channel on it. In similar way with the abovementioned standing nanorod assembly HMM, multiple highly dispersive bulk plasmon modes with extremely large field confinement are supported in the stratified HMM region from the visible to the near infrared. The differences from the above nanorod HMMs are as follows. At first, the grating coupled stratified HMM biosensor by Sreekanth et al. [136,137] can operate from the visible to the near infrared as shown by multiple guided mode resonances in Figure 9d. Secondly, as it utilizes grating coupling technique rather than ATR, the setup is more miniaturized with excellent performance. Couplings between incident broadband source and the bulk plasmon modes are achieved by the two-dimensional grating rather than using a prism. The grating enables large amount of high-k diffraction in the hyperbolic region. Their device exhibits records of spectral sensitivity reaching 30,000 nm/RIU with FOM of 590 and angular sensitivity reaching 7000 degree/RIU which largely exceeds the conventional plasmonic and nanophotonic biosensor platforms based on the ATR, localized surface plasmon resonances, and extraordinary transmissions. Moreover, they report the ability to detect extremely low molecular weight (244 Da) biomolecules at picomolar concentrations.

### 2.4. Sensors Based on Holographic Phase Gradient

Since concepts of holographic phase jump and generalized Snell’s law were suggested by N. Yu et al. [138], much attention has been paid mainly on various MS schemes for ultrathin flat optic components aiming at exotic light bending [138,139,140], light focusing [50,141,142,143] and holographic imaging [144,145,146]. Equations (5) and (6) denote analytic transmission and reflection principles, respectively.

(5) $$n_{t}\sin\theta_{t}-n_{i}\sin\theta_{i}=\frac{1}{k_{0}}\frac{d\Phi }{dx},$$

(6) $$\sin\theta_{r}-\sin\theta_{i}=\frac{1}{n_{i}k_{0}}\frac{d\Phi }{dx},$$

where *n_t_*, *n_i_*, *θ_t_*, *θ_i_*, *k*_0_, and Φ denote RI of output transmission region, RI of input region, transmission angle, incident angle, wavenumber in vacuum, and optical phase jump at MS depending on location. Compared to original Snell’s law of refraction, newly generalized one considers abrupt jump of optical phase imparted by ultrathin optical MS at the interface of two optical media (*n_t_* and *n_i_*). Thus, generalized Snell’s law implies that various bending, focusing and imaging functionalities are available by designing HPG on MSs. Pancharatnam Berry phase with anisotropic [137,138,139,140,141,142,143,144,145,146,147,148,149,150] and multi-modal V-shaped MAs [138] have been utilized for engineering spatial HPG.

**Figure 10:**
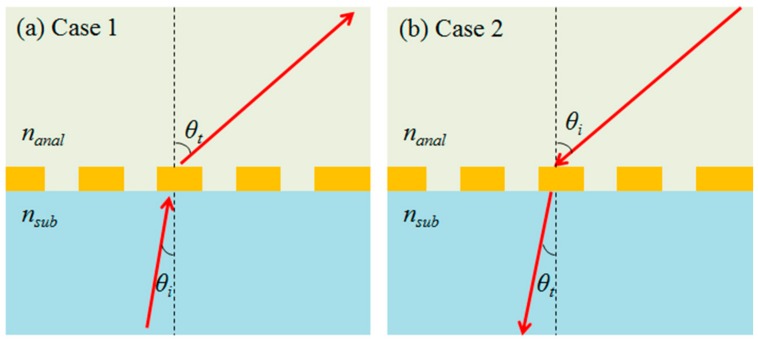
The simplest schemes for refractive index sensing with an HPG MS and tunable refractions when an input beam is incident from (**a**) a substrate side and (**b**) a superstrate analyte side. *n_anal_*, *n_sub_*, *θ_i_*, and *θ_t_* denote refractive index of analyte layer, refractive index of substrate, incident angle of an input beam, and refraction angle of a transmitted beam. The yellow patterns represent an arbitrary HPG MS.

This anomalous principle with large tunability can also be applied to various methods of RI sensing. We can consider the simplest configurations to sense refractive indices of analytes by monochromatic coherent light and its refraction angle controlled by incidence angle and HPG. As depicted in Figure 10a,b, the schemes consist of substrate, an HPG imparted MS, and an analyte layer. Figure 10a describes the case when an input beam is incident from substrate side and refracted into an analyte layer. Variation of *n_anal_* is detected by followed change of *θ_t_*, which can be freely designed based on Equation (5). Figure 10b describes the case when an input beam is incident from anlayte layer and refracted into a substrate side. Using definition of sensitivity based on resonant nanophotonic sensors, angular sensitivities of these non-resonant configurations can be analytically derived as shown in Equation (7). The first and second rows in the right hand side of Equation (7) denote the cases of Figure 10a (the case 1) and 10b (the case 2), respectively.

(7) $$S=\frac{\partial \theta_{t}}{\partial n_{anal}}=\{\begin{array}{ll} -(\alpha^{3}/n_{anal}^{2})\sqrt{1-{\left(\alpha /n_{anal}\right)}^{2}},\;(\alpha =n_{sub}\sin\theta_{i}+\frac{1}{k_{0}}\frac{d\Phi }{dx}) & \mathrm{for}\;\mathrm{case}\;1\;\\ \frac{\sin\theta_{i}}{n_{sub}}\sqrt{1-(n_{anal}\sin\theta_{i}-\frac{1}{k_{0}}\frac{d\Phi }{dx}{)}^{2}/n_{sub}{}^{2}}, & \mathrm{for}\;\mathrm{case}\;2 \end{array}$$

(8) $$S_{\max}=\{\begin{array}{ll} {\frac{\partial \theta_{t}}{\partial n_{anal}}|}_{\frac{1}{k_{0}}\frac{d\Phi }{dx}=\pm \frac{\sqrt{3}}{2}n_{anal}-n_{i}\sin\theta_{i}}=\frac{3\sqrt{3}}{16}n_{anal}\;[\deg/\mathrm{RIU}], & \mathrm{for}\;\mathrm{case}\;1\\ {\frac{\partial \theta_{t}}{\partial n_{anal}}|}_{\frac{1}{k_{0}}\frac{d\Phi }{dx}=n_{anal}\sin\theta_{i}}=\frac{\sin\theta_{i}}{n_{sub}}\;[\deg/\mathrm{RIU}]. & \mathrm{for}\;\mathrm{case}\;2 \end{array}$$

As shown in Equation (7), angular sensitivity is highly dependent on an incident angle from which side light impinges, and a designed HPG on MS. Assuming certain incidence conditions and specific analyte of interest, the maximal angular sensitivity of the HPG MS sensor is analytically derived as written in Equation (8). Even though the sensitivities of two configurations are not in comparable order with conventional resonant sensors, it is worth noting that sensing RI is possible utilizing a monochromatic laser light and a camera to measure variation of refracting direction rather than using conventional and bulkier setup with a broadband light source and an optical spectrometer. Although the simplest configurations are presented here for proof-of-concept with poor sensitivities, if configurations with high-index or resonant MAs are constructed with an HPG MS, angular and spectral sensitivities can be highly improved. Moreover, it would be also possible to design novel MS sensors with broadband input source if the wavelength dependent properties of HPG and refraction angle are utilized with resonant MAs.

## 3. Sensing Light Properties with MMs and MSs

### 3.1. Sensing Polarization State of Light: Compact Polarization Resolving

#### 3.1.1. Resolving Photonic Spin with Helical Beam Splitters and Circular Polarizers

The polarization resolving is highly desirable in the field of optical MMs and flat optics nowadays. To achieve diverse functionalities in compact and light volume, carrying and manipulating optical information in polarization-dependent manners are studied thoroughly for various flat optic components [48,49,50]. Especially, the two orthonormal photonic spins (PSs) have attracted enormous interest in the optical engineering society. Photonic spin is also known as circular polarization, optical handedness, and light helicity. The two orthogonal polarization states with opposite photonic spins have been widely applied to image sensing systems for contrast enhancement [151], planetary detection of chiral signatures [152], augmented/virtual reality [153], and microscopy of inherently chiral biomolecules [152,154,155]. Hence, resolving and decomposing PS composition of certain polarized beam is a significant goal for practical applications.

Recently, optical Pancharatnam Berry phase, also known as geometric phase, has been thoroughly studied to decompose light based on PS-dependent wavefront shaping [147,148,149,150]. Individual anisotropic MAs including nanoslit and nanopillar made of noble metals or high-index dielectrics act as nanoscale wave-plates so that PS-dependent optical geometric phase is induced by individual local rotation angle of a MA. A single MS with optical geometric phase elements can impart two different HPGs for the two different PSs as presented in the Equation (9) [48].

(9) $$E_{t}^{R/L}=\frac{t_{0}+t_{e}}{2}E_{I}^{R/L}+\frac{t_{0}-t_{e}}{2}\exp(\mp i2\alpha )E_{I}^{L/R}$$

**Figure 11:**
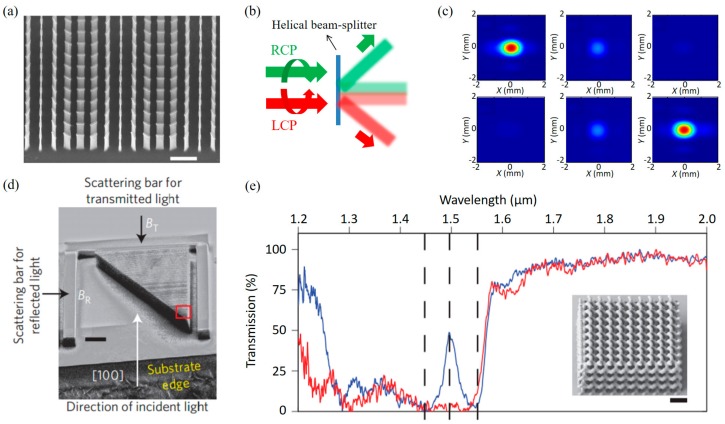
(**a**) SEM image; and (**b**) schematic illustration of helical beam-splitter demonstrated by M. Khorasaninejad and K. B. Crozier [156]. Scale bar, 1 μm; (**c**) Captured CCD images of diffraction. The first and second rows present LCP and RCP cases. The first, second, and third columns present −1, zero, and +1 order of diffractions. (**d**) Oblique SEM image of gyroid crystal metamaterial (GCMM) prism [157]. Scale bar, 20 μm; (**e**) Transmission spectra of GCMM for two different optical handedness. Red and blue lines denote RCP and LCP incidences, respectively. Inset image represents SEM image of the GCMM. Scale bar, 2 μm. Reprinted by permission from Macmillan Publishers Ltd.: (Nature Communications) (Reference [156]), copyright (2014) and (Nature Photonics) (Reference [157]), copyright (2013).

In the above equation, the first term of the right-hand side corresponds to co-polarized transmission while the second term represents cross-polarized transmission with additional geometric phase term determined to the two times of rotation angle (*α*). Based on the Equation (9), two different linear HPGs and distinct 1st order diffractions can be acheved according to the PS of transmitted light. In 2014, Khorasaninejad and Crozier proposed silicon nanofin MS (See Figure 11a,b) as a miniature helical beam-splitter operating in the near-infrared using aforementioned principle of geometric phase [156]. Owing to large scattering cross-section and aspect ratio of silicon nanofin, a high extinction ratio (ER) reaching 50 is achieved. Moreover, as optical geometric phase is dominated by local rotation angle of a MA and independent of wavelength, broadband operation of helical beam-splitting is possible. The main disadvantage of the device is that there is always large amount of 0th order unfiltered diffraction without certain handedness as shown in Figure 11c.

In 2013, Turner et al. proposed different type of helical beam-splitter using polymer (*n* = 1.48) based gyroid crystal MM (GCMM) made by direct laser writing (DLW) [157]. GCMM shows PS-dependent three-dimensional circulating currents with strong bianisotropic effects. By shaping GCMM into prism-like shape in millimeter scale (Figure 11d), LCP and RCP compositions are decomposed with moderate ERs (5.12 dB in transmission and 3.46 dB in reflection) in the telecom wavelength (Figure 11e). Even though the GCMM helical beam-splitter is difficult to fabricate and has relatively low ERs compared to abovementioned nanofin beam-splitter, it is noteworthy that the GCMM shows chiral resonances without lossy and opaque materials.

**Figure 12:**
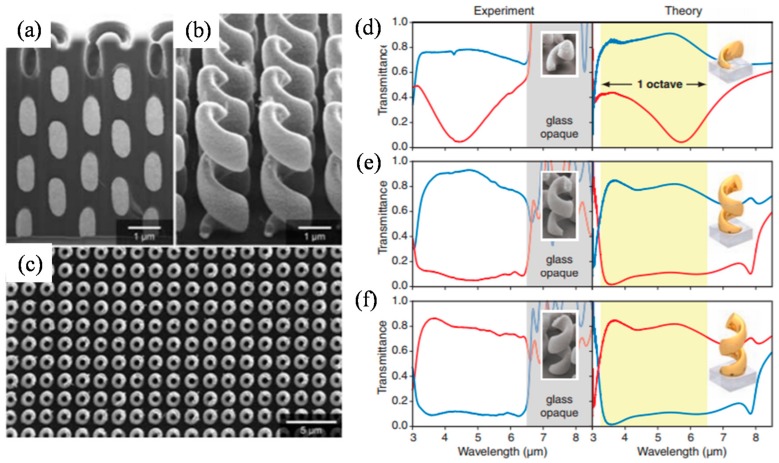
(**a**–**c**) SEM images of helix MM circular polarizer (HMCP) made by J. K. Gansel et al. [158]. Transmission spectra of HMCP with: (**d**) one; (**e**) two; and (**f**) three pitches of helices. Blue and red curves represent RCP and LCP, respectively. From Reference [158]. Reprinted with permission from AAAS.

While the helical beam-splitters decompose two PSs by imparting different momenta on them, circular polarizers have been proposed for filtering transmission of certain target PS. There are three popular types of circular polarizers using optical MMs and MSs. The first one is metallic helix MM (See Figure 12a–c) first proposed by Gansel et al. in 2009 [158]. Metallic helix structure shows highly asymmetric transmission according to optical handedness of incident light owing to strong bianisotropic effects with circulating currents along helices. The rotating directions of helix structures determine target photonic spin to transmit or reflect. The advantages of the structure are broad bandwidth and moderate ERs up to about 9 (See Figure 12d–f). There have been many efforts to develop fabrication processes for complex helix structures using DLW [158,159], DNA based self-assembly [160], focused ion beam induced deposition [161], glancing-angle deposition [162,163], or tomographic rotatory growth [164].

**Figure 13:**
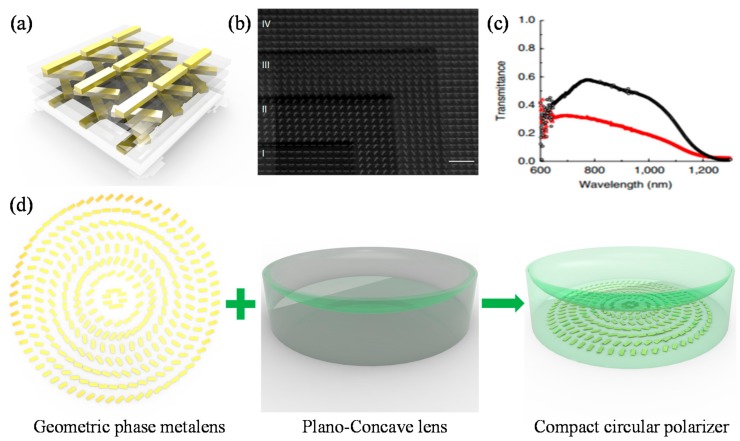
(**a**) Schematic illustration; and (**b**) ion beam assisted SEM image of rotationally stacked nanorod MM [165]; (**c**) Optical transmission spectra for LCP and RCP incidences. Black and Red curves refer to RCP and LCP, respectively; (**d**) Schemes of a geometric phase metalens, plano-concave dielectric lens, and hybrid compact circular polarizer [167]. Reprinted by permission from Macmillan Publishers Ltd.: (Nature Communications) (Reference [165]), copyright (2012).

The second type of circular polarizer is rotationally stacked nanorod metasurface (RSNM) [165,166]. To overcome fabrication difficulties in submicron scale and achieve visible and near-infrared operation, the gold RSNM has been demonstrated with sequential electron-beam lithography as shown in Figure 13a,b. Based on similar physical origin with helices, strong bianisotropic interactions between rotationally stacked nanorods exhibit broad helicity-dependent transmission with moderate extinction ratio up to 8 (Figure 13c).

The third type based on geometric phase meta-lens was demonstrated by Ke et al. in 2016 [167]. They incorporated a gold nanorod visible meta-lens using geometric phase and a bulk-optic plano-concave lens as shown in Figure 13d. While meta-lens imparts converging and diverging quadratic wavefront on the two distinct PSs, the plano-concave bulk lens imparts diverging quadratic wavefront for both PSs. Hence, only LCP light transmits and RCP light diverges after passing through the hybrid compact circular polarizer. The plano-concave bulk lens was fabricated using irradiation of femtosecond laser. The irradiated fused glass was decomposed into porous glass so that its RI is controlled by the laser intensity. Even though the fabrication process is complicated, broadband operation is possible owing to the broad bandwidth of geometric phase.

#### 3.1.2. Resolving Arbitrary Polarization State with MSs: Ultracompact Polarimetry

Complete resolving of arbitrary polarization state is a more complex and significant goal compared to PS resolving. As a single polarization state is described by a set of four Stokes parameters in Poincare space, conventional polarimetry setups have been bulky and complex for multiple and interferometric measurements of optical signals. Hence, MSs have been proposed for resolving of arbitrary state of polarization in ultracompact area. Wen et al. measured ellipticity and helicity simultaneously with an HPG MS using optical geometric phase and a CCD camera [168]. However, their device cannot characterize every polarization state on the surface of Poincare sphere.

**Figure 14:**
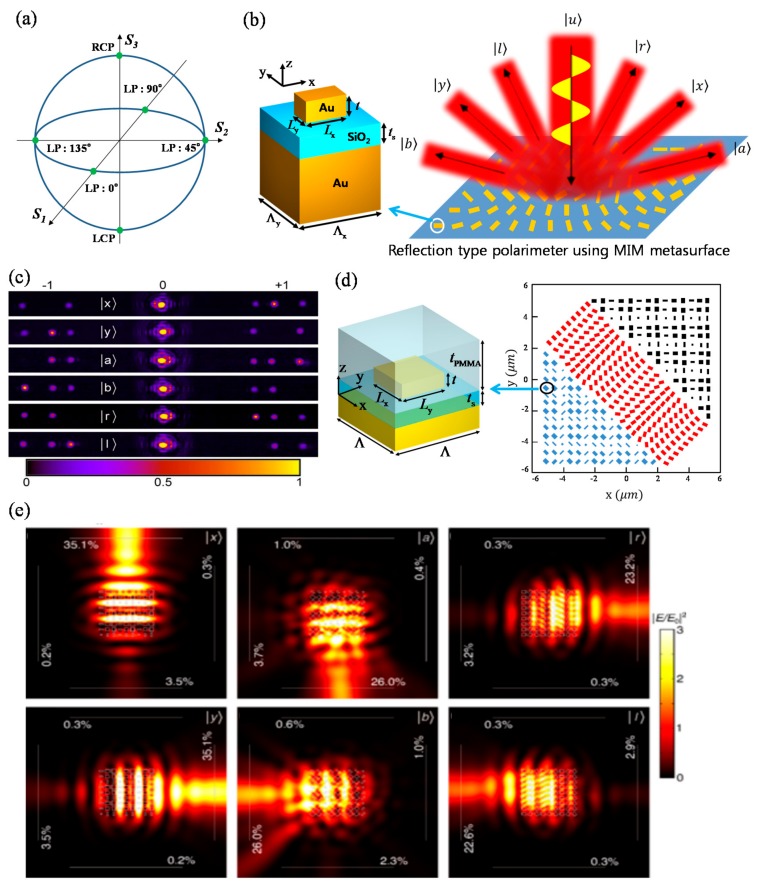
(**a**) Poincare sphere with four linear polarizations (LPs) and left circular polarization (LCP) and right circular polarization (RCP) as the six green dots; (**b**) Schematic illustration of reflection-type MIM MS polarimeter by A. Porst et al. [169]; (**c**) Far-field reflection images for the six different polarization states; (**d**) Scheme of reflective MIM MS in-plane polarimeter [170]; (**e**) Numerically calculated SPP excitation patterns when the three different unit-cells of MIM in-plane polarimeter are illuminated by the six differently polarized light. The first, second, and third column images correspond to the cases of the unit-cells for the black, blue, and red regions in (**d**). The upper right kets written in the six images denote polarizations state of incident beam. From Reference [169]. The part (**e**) is reprinted by permission from Reference [170].

Recently, Pors et al. [169] proposed complete characterizations of Stokes parameters for near-infrared wavelengths with reflection-type metal-insulator-metal (MIM) MS as shown in Figure 14b. They used optical geometric phase and additional phase dependent on dimensions of gold nanoantennas in the reflective MS. Their device scatters light in the six different directions (Figure 14b,c). Light intensities reflected in each direction imply quantitative compositions of two orthogonal and linear polarizations, two orthogonal and linear polarizations which are rotated in 45 degrees from the former set, and two orthogonal circular polarizations. Simultaneous measurements of the six reflected intensities resolve arbitrary polarization state. In 2016, they proposed the similar device for in-plane polarimeter (Figure 14d) using SPP excitation instead of far-field reflection [170]. Figure 14e shows that the propagations of SPPs are largely switched according to the polarization state of input beam incident on the distributed MIM nanoantennas depicted in Figure 14d. In Figure 14e, the first, second, and third columns correspond to the cases when the input normal beam is incident on the unit-cells of the black, blue, and red regions depicted in Figure 14d. The six polarization states of incident light are described in the six images of Figure 14d, respectively.

**Figure 15:**
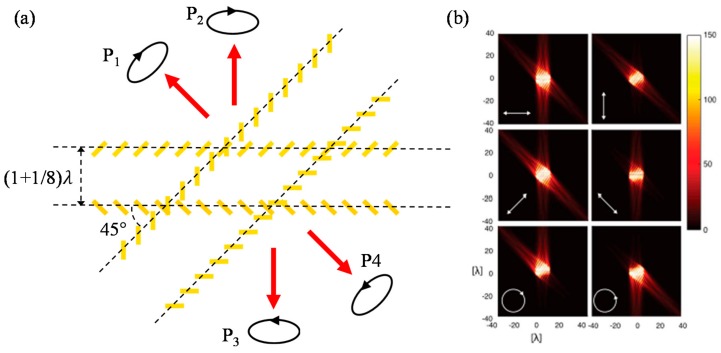
(**a**) Unit cell scheme of polarimeter designed by Muller et al. [171] (**b**) presents far-field scattering in case of the six distinct polarizations, which are described with the white arrows in the plots. From Reference [171].

In 2016, Muller et al. proposed highly improved MS based polarimeter for nondestructive, transmission-type in-line polarimetery with a single MS and a CCD camera [171]. By placing interlocked gold nanorod periodically, they measured quantities of two different elliptical polarizations at first. Then, by multiplexing another periodic interlocked gold nanorod array in plane, they measured quantitative compositions of two other elliptical polarizations for complete determination of Stokes paramters (Figure 15a,b). It showed a great possibility of practical usage as a miniaturized integrable polarimeter which is essential in various complex systems.

### 3.2. Optical Spectrum Analysis with MMs and MSs: Ultracompact Spectroscopy

Spectroscopy is a scientific measurement technique for the analysis of the interaction between matter and EM radiation with different components of EM spectrum. It can analyze light by breaking it down into several frequencies using dispersion phenomenon, which is the basic and fundamental principle in optics. Spectrometer has been widely used in environmental science to probe and determine water properties such as acidity in samples, biomedical science as diagnostic and therapeutic applications, and astronomy for calculating the relative velocities of supernovae and galaxies by measuring red-shift.

**Figure 16:**
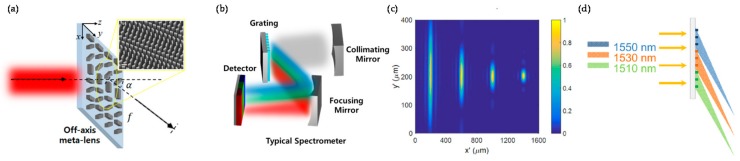
(**a**) Schematic illustration of off-axis meta-lens. The inset is SEM image of meta-lens; (**b**) Schematic configuration of operation of a conventional grating-based spectrometer. Dispersive and focusing elements are separated; (**c**) Phase profiles of meta-lenses along both *x*- and *y*-axes; (**d**) Three meta-lenses are stitched together. Each covers a bandwidth of interest without interfering with other bandwidth. Reprinted with permission from Reference [173]. Copyright (2016) American Chemical Society.

Conventional optical spectrometer (OS) is designed to decompose input beam spatially using achromatic focusing mirror, dispersive optical grating turret, and detector [172]. For sufficient grating dispersion, it is absolutely necessary to secure a large spatial separation with long optical distance, and that is a factor that hinders miniaturization of bulky configuration. However, the advent of MS concept has enabled the miniaturization of conventional OS system which consists of ultrathin and super-dispersive MS and a detecting camera [172,173,174]. Recently, F. Capasso group proposed abovementioned two-component OS system operating in the visible [172] and the near-infrared wavelengths [173]. They designed off-axis meta-lenses to focus incident light at an angle α as shown in Figure 16a. Their works were based on the principle of geometric phase, which can control optical phase fully by rotating the orientation of nanorods. Phase difference to achieve constructive interference at a focal point was compensated by this method. At this point, the required phase profile depends on wavelength of incidence, thus the focal point moves owing to chromatic aberration which occurs by changing the wavelength. They calculated the spectral resolution (δλ) of the meta-lens, the quantification of OS performance. The result is given by

(10) $$\delta \lambda =\frac{\Delta \lambda }{f\Delta \alpha }\times \frac{0.61\lambda }{NA}=\frac{\Delta \lambda }{f\left\{\sin^{-1}\left[\left(1+\frac{\Delta \lambda }{\lambda_{d}}\sin\left(\alpha \right)\right)\right]-\alpha \right\}}\times \frac{0.61\lambda }{NA}$$

where *Δα* is the change in the focusing angle due to a small change of the incident wavelength (Δ*λ*), f is focal length, and *λ_d_* is the design wavelength. Also, *NA* is numerical aperture and it determines the minimum resolvable wavelength difference in the focal plane based on the Rayleigh criterion (0.61*λ*/*NA*). According to Reference [173], meta-lenses-based OS achieved the detector-limited spectral resolution up to 0.3 nm and a total working wavelength range exceeding 170 nm for a beam propagation length of only a few cm. It showed similar performance and functionality with ultra-compact configuration compared with conventional grating-based OS. It is expected that the range of its potential applications would be far broader if large-scale nanofabrication problems are solved.

## 4. Conclusions

In this paper, various photonic MMs and MSs for RI sensing and light property sensing are reviewed and categorized according to their objectives and working principles. Table 2 and Table 3 show RI sensors, PS resolving devices, polarimeters, and spectrometers based on MMs and MSs. This review would help to choose appropriate structures and physical principles for certain RI sensing objects. Moreover, it can be fruitful to potential compact and high-performance platforms of various light property analyzer which can be easily integrated to electronic smart devices.

## Figures and Tables

**Table 1:** Various types of nanostructures for SERS and their features.

Type	Features	References
Tip	Strong localized surface plasmon resonance (LSPR) generation by tip-enhanced Raman spectroscopy (TERS) Diffraction-limited imaging with scanning probe microscopy (SPM) Relatively hard to fabricate and reproduce	[59,60,61,62]
Gap	High confinement of electromagetic field Single molecule detection using nanogaps Relatively easy fabrication (E-beam lithography, focused ion beam)	[65,66,67]
Pore	Easily combined with array structure for extraordinary optical transmission (EOT) Easily tunable geometric parameters (period, size)	[63,64]
Sphere	Relatively easy to fabricate (solution-based batch processing) High EFs and stability (Ag film over nanosphere substrate) High reactivity with large surface area	[70,71]

**Table 2:** Performance of MMs-based RI sensors.

Structure Type	Wavelength (or Frequency)	Detection RI Range	EF/Sensitivity	FOM	Q-Factor	References
**SERS-Based Sensors**						
SRR	vis-NIR	water, K^+^ buffer	EF: 6.5 × 10^7^ Sensitivity: 378~655 nm/RIU			[85]
Disk	vis-NIR	water	EF: 7.8 × 10^7^			[87]
Disk	vis-NIR	benzenethiol solution	EF: 8.4 × 10^7^			[88]
Grating-type patterned porous gold substrate	vis-NIR	benzenethiol solution	EF: 5.0 × 10^7^			[91]
Fano resoanance-based sensors						
ASR	~0.5 THz		5.7 × 10^4^ nm/RIU		28	[113]
SRR	~ 1000 nm	2-naphthalenoethiol	436 nm/RIU			[84]
Cut-out structure	~2000 nm	water, glucose solution (1.333~1.372)	588 nm/RIU	5.3	28	[25]
Asymmetric double bars	~1000 nm				7.34	[117]
Crosses	~1.2 THz	Isopropanol, glycerin, paraffin, ethanol, butanol, rapeseed oil, cyclohexane, NEP	0.4 THz/μm			[119]
Silver nanocube	~480 nm		~953 nm/RIU	12~20		[122]
Gold on photoresist pillar	~1000 nm	cytochrome *c* (1.333~1.417)	1010 nm/RIU	108		[123]
Single nanoslit with periodic grooves	~810 nm	1.333–1.3415	615 nm/RIU	48		[125]
Capped gold nanoslit	~800 nm	1.333–1.384	926 nm/RIU	252		[126]
HMMs-based sensors						
Standing nanorods	~1200 nm		30,000 nm/RIU	330		[135]
Stratified HMMs	450~1300 nm	1.3330~1.3336	30,000 nm/RIU 7000 nm/RIU	590		[136,137]

**Table 3:** Flat optic devices based on MMs and MSs for characterization of light properties.

Purpose Features			Wavelength	Performance	References
Polarization resolving	Photonic spin resolving	Helical beam splitter			
		Geometric phase based Gyroid MM based	NIR Telecom.	ER ~50 ER ~3.25	[156] [157]
		Circular polarizer			
		Helix MM based nanorod MM based Geometric phase based	IR visible~NIR visible	ER ~9 ER ~8	[158,159,160,161,162,163,164] [165,166] [167]
	Polarimetry (Full Stokes parameters)	Reflection-type Transmission-type	NIR Telecom	BW ~ 300 nm BW > 65 nm	[169,170] [171]
Spectroscopy		Geometric phase based	Visible NIR	δλ ~ 0.3 nm δλ ~ 0.2 nm	[172] [173]

## References

[1] Cai W., Shalaev V. (2010). Optical Metamaterials: Fundamentals and Applications.

[2] Zheludev N.I., Kivshar Y.S. (2012). From metamaterials to metadevices. Nat. Mater..

[3] Meinzer N., Barnes W.L., Hooper I.R. (2014). Plasmonic meta-atoms and metasurfaces. Nat. Photonics.

[4] Poddubny A., Iorsh I., Belov P., Kivshar Y. (2013). Hyperbolic metamaterials. Nat. Photonics.

[5] Pendry J.B. (2000). Negative refraction makes a perfect lens. Phys. Rev. Lett..

[6] Shalaev V.M. (2007). Optical negative-index metamaterials. Nat. Photonics.

[7] Pendry J.B. (2004). A chiral route to negative refraction. Science.

[8] Luo C., Johnson S.G., Joannopoulos J.D., Pendry J.B. (2002). All-angle negative refraction without negative effective index. Phys. Rev. B.

[9] Shelby R.A., Smith D.R., Schultz S. (2001). Experimental verification of a negative index of refraction. Science.

[10] Shalaev V.M., Cai W., Chettiar U.K., Yuan H.K., Sarychev A.K., Drachev V.P., Kildishev A.V. (2005). Negative index of refraction in optical metamaterials. Opt. Lett..

[11] Valentine J., Zhang S., Zentgraf T., Ulin-Avila E., Genov D.A., Bartal G., Zhang X. (2008). Three-dimensional optical metamaterial with a negative refractive index. Nature.

[12] Landy N.I., Sajuyigbe S., Mock J.J., Smith D.R., Padilla W.J. (2008). Perfect metamaterial absorber. Phys. Rev. Lett..

[13] Liu X., Starr T., Starr A.F., Padilla W.J. (2010). Infrared spatial and frequency selective metamaterial with near-unity absorbance. Phys. Rev. Lett..

[14] Hao J., Wang J., Liu X., Padilla W.J., Zhou L., Qiu M. (2010). High performance optical absorber based on a plasmonic metamaterial. Appl. Phys. Lett..

[15] Hedayati M.K., Javaherirahim M., Mozooni B., Abdelaziz R., Tavassolizadeh A., Chakravadhanula V.S.K., Zaporojtchenko V., Strunkus T., Faupel F., Elbahri M. (2011). Design of a perfect black absorber at visible frequencies using plasmonic metamaterials. Adv. Mater..

[16] Liu N., Mesch M., Weiss T., Hentschel M., Giessen H. (2010). Infrared perfect absorber and its application as plasmonic sensor. Nano Lett..

[17] Hao J., Zhou L., Qiu M. (2011). Nearly total absorption of light and heat generation by plasmonic metamaterials. Phys. Rev. B.

[18] Feng S., Halterman K. (2012). Coherent perfect absorption in epsilon-near-zero metamaterials. Phys. Rev. B.

[19] Søndergaard T., Novikov S.M., Holmgaard T., Eriksen R.L., Beermann J., Han Z., Bozhevolnyi S.I. (2012). Plasmonic black gold by adiabatic nanofocusing and absorption of light in ultra-sharp convex grooves. Nat. Commun..

[20] Malassis L., Massé P., Tréguer-Delapierre M., Mornet S., Weisbecker P., Barois P., Simovski R., Kravets V.G., Grigorenko A.N. (2014). Topological darkness in self-assembled plasmonic metamaterials. Adv. Mater..

[21] Cao T., Wei C., Simpson R.E., Zhang L., Cryan M.J. (2014). Broadband polarization-independent perfect absorber using a phase-change metamaterial at visible frequencies. Sci. Rep..

[22] Fountaine K.T., Cheng W.-H., Bukowsky C.R., Atwater H.A. (2016). Near-unity unselective absorption in sparse InP nanowire arrays. ACS Photonics.

[23] Liu N., Langguth L., Weiss T., Kästel J., Fleischhauer M., Pfau T., Giessen H. (2009). Plasmonic analogue of electromagnetically induced transparency at the Drude damping limit. Nat. Mater..

[24] Zhang S., Genov D.A., Wang Y., Liu M., Zhang X. (2008). Plasmon-induced transparency in metamaterials. Phys. Rev. Lett..

[25] Liu N., Weiss T., Mesch M., Langguth L., Eigenthaler U., Hirscher M., Sönnichsen C., Giessen H. (2009). Planar metamaterial analogue of electromagnetically induced transparency for plasmonic sensing. Nano Lett..

[26] Pfeiffer C., Grbic A. (2013). Metamaterial Huygens’ surfaces: Tailoring wave fronts with reflectionless sheets. Phys. Rev. Lett..

[27] Monticone F., Estakhri N.M., Alù A. (2013). Full control of nanoscale optical transmission with a composite metascreen. Phys. Rev. Lett..

[28] Decker M., Staude I., Falkner M., Dominguez J., Neshev D.N., Brener I., Pertsch T., Kivshar Y.S. (2015). High-efficiency dielectric Huygens’ surfaces. Adv. Opt. Mater..

[29] Liu Z., Lee H., Xiong Y., Sun C., Zhang X. (2007). Far-field optical hyperlens magnifying sub-diffraction-limited objects. Science.

[30] Jacob Z., Alekseyev L.V., Narimanov E. (2006). Optical hyperlens: Far-field imaging beyond the diffraction limit. Opt. Express.

[31] Rho J., Ye Z., Xiong Y., Yin X., Liu Z., Choi H., Bartal G., Zhang X. (2010). Spherical hyperlens for two-dimensional sub-diffractional imaging at visible frequencies. Nat. Commun..

[32] Wu C., Khanikaev A.B., Shvets G. (2011). Broadband slow light metamaterial based on a double-continuum Fano resonance. Phys. Rev. Lett..

[33] Manjappa M., Chiam S.Y., Cong L., Bettiol A.A., Zhang W., Singh R. (2015). Tailoring the slow light behavior in terahertz metasurfaces. Appl. Phys. Lett..

[34] Tsakmakidis K.L., Boardman A.D., Hess O. (2007). ‘Trapped rainbow’ storage of light in metamaterials. Nature.

[35] Schurig D., Mock J.J., Justice B.J., Cummer S.A., Pendry J.B., Starr A.F., Smith D.R. (2006). Metamaterial electromagnetic cloak at microwave frequencies. Science.

[36] Chen H., Wu B.I., Zhang B., Kong J.A. (2007). Electromagnetic wave interactions with a metamaterial cloak. Phys. Rev. Lett..

[37] Silveirinha M.G., Alù A., Engheta N. (2007). Parallel-plate metamaterials for cloaking structures. Phys. Rev. E.

[38] Ergin T., Stenger N., Brenner P., Pendry J.B., Wegener M. (2010). Three-dimensional invisibility cloak at optical wavelengths. Science.

[39] Gabrielli L.H., Cardenas J., Poitras C.B., Lipson M. (2009). Silicon nanostructure cloak operating at optical frequencies. Nat. Photonics.

[40] Alù A., Engheta N. (2008). Multifrequency optical invisibility cloak with layered plasmonic shells. Phys. Rev. Lett..

[41] Chung T., Lee S.-Y., Song E.Y., Chun H., Lee B. (2011). Plasmonic nanostructures for nano-scale bio-sensing. Sensors.

[42] Roh S., Chung T., Lee B. (2011). Overview of the characteristics of micro- and nano-structured surface plasmon resonance sensors. Sensors.

[43] Chen T., Li S., Sun H. (2012). Metamaterials application in sensing. Sensors.

[44] Khanikev A.B., Wu C., Shevets G. (2013). Fano-resonant metamaterials and their applications. Nanophotonics.

[45] Luk’yanchuk B., Zheludev N.I., Maier S.A., Halas N.J., Nordlander P., Giessen H., Chong C.T. (2010). The Fano resonance in plasmonic nanostructures and metamaterials. Nat. Mater..

[46] Dayal G., Ramakrishna S.A. (2013). Design of multi-band metamaterial perfect absorbers with stacked metal–dielectric disks. J. Opt..

[47] Watts C.M., Liu X., Padilla W.J. (2012). Metamaterial electromagnetic wave absorbers. Adv. Opt. Mater..

[48] Yu N., Capasso F. (2014). Flat optics with designer metasurfaces. Nat. Mater..

[49] Jahani S., Jacob Z. (2016). All-dielectric metamaterials. Nat. Nanotechnol..

[50] Khorasaninejad M., Chen W.T., Devlin R.C., Oh J., Zhu A.Y., Capasso F. (2016). Metalenses at visible wavelengths: Diffraction-limited focusing and subwavelength resolution imaging. Science.

[51] Ding F., Wang Z., He S., Shalaev V.M., Kildishev A.V. (2015). Broadband high-efficiency half-wave plate: A supercell-based plasmonic metasurface approach. ACS Nano.

[52] Haynes C.L., McFarland A.D., Van Duyne R.P. (2005). Surface-enhanced Raman spectroscopy. Anal. Chem..

[53] Fleischmann M., Hendra P.J., McQuillan A.J. (1974). Raman spectra of pyridine absorbed at a silver electrode. Chem. Phys. Lett..

[54] Jeanmaire D.L., Van Duyne R.P. (1977). Surface Raman spectroelectrochemistry: Part I. Heterocyclic, aromatic, and aliphatic amines absorbed on the anodized silver electrode. J. Electroanal. Chem..

[55] Albrecht M.G., Creighton J.A. (1977). Anomalously intense Raman spectra of pyridine at a sliver electrode. J. Am. Chem. Soc..

[56] Maier S.A. (2007). Plasmonics: Fundamentals and Applications.

[57] Maier S.A. (2006). Plasmonic field enhancement and SERS in the effective mode volume picture. Opt. Express.

[58] Le Ru E.C., Blackie E., Meyer M., Etchegoin P.G. (2007). Surface enhanced Raman scattering enhancement factors: A comprehensive study. J. Phys. Chem. C.

[59] Liu G.L., Lu Y., Kim J., Doll J.C., Lee L.P. (2005). Magnetic nanocrescents as controllable surface-enhanced Raman scattering nanoprobes for biomolecular imaging. Adv. Mater..

[60] Zhang W., Xudong, Yeo B.-S., Schmid T., Hafner C., Zenobi R. (2007). Nanoscale roughness on metal surfaces can increase tip-enhanced Raman scattering by an order of magnitude. Nano Lett..

[61] Lindquist N.C., Nagpal P., Lesuffleur A., Norris D.J., Oh S.-H. (2010). Three-dimensional plasmonic nanofocusing. Nano Lett..

[62] Guieu V., Talaga D., Servant L., Sojic N., Lagugné-Labarthet F. (2009). Multitip-localized enhanced Raman scattering from a nanostructured optical fiber array. J. Phys. Chem. C.

[63] Baumberg J.J., Kelf T.A., Sugawara Y., Cintra S., Abdelsalam M.E., Bartlett P.N., Russell A.E. (2005). Angled-resolved surface-enhanced Raman scattering on metallic nanostructured plasmonic crystals. Nano Lett..

[64] Yu Q., Guan P., Qin D., Golden G., Wallace P.M. (2008). Inverted size-dependence of surface-enhanced Raman scattering on gold nanohole and nanodisk arrays. Nano Lett..

[65] Im H., Bantz K.C., Lindquist N.C., Haynes C.L., Oh S.-H. (2010). Vertically oriented sub-10-nm plasmonic nanogap arrays. Nano Lett..

[66] Gopinath A., Boriskina S.V., Premasiri W.R., Ziegler L., Reinhard B.M., Dal Negro L. (2009). Plasmonic nanogalaxies: Multiscale aperiodic arrays for surface-enhanced Raman scattering. Nano Lett..

[67] Lee B., Lee I.-M., Kim S., Oh D.-H., Hesselink L. (2010). Review on subwavelength confinement of light with plasmonics. J. Mod. Opt..

[68] Lim D.-K., Jeon K.-S., Hwang J.-H., Kim H., Kwon S., Suh Y.D., Nam J.-M. (2011). Highly uniform and reproducible surface-enhanced Raman scattering from DNA-tailorable nanoparticles with 1-nm interior gap. Nat. Nanotechnol..

[69] Lim D.-K., Jeon K.-S., Kim H.M., Nam J.-M., Suh Y.D. (2010). Nanogap-engineerable Raman-active nanodumbbells for single-molecule detection. Nat. Mater..

[70] Wustholz K.L., Brosseau C.L., Casadio F., Van Duyne R.P. (2009). Surface-enhanced Raman spectroscopy of dyes: From single molecules to the artists’ canvas. Phys. Chem. Chem. Phys..

[71] Liu X., Sun C.-H., Linn N.C., Jiang B., Jiang P. (2009). Wafer-scale surface-enhanced Raman scattering substrates with highly reproducible enhancement. J. Phys. Chem. C.

[72] Lin H., Mock J., Smith D., Gao T., Sailor M.J. (2004). Surface-enhanced Raman scattering from silver-plated porous silicon. J. Phys. Chem. B.

[73] Pendry J.B., Holden A.J., Robbins D.J., Stewart W.J. (1999). Magnetism from conductors and enhanced nonlinear phenomena. IEEE Trans. Microw. Theory Tech..

[74] Soukoulis C.M., Linden S., Wegener M. (2007). Negative refractive index at optical wavelengths. Science.

[75] Smith D.R., Padilla W.J., Vier D.C., Nemat-Nasser S.C., Schultz S. (2000). Composite medium with simultaneously negative permeability and permittivity. Phys. Rev. Lett..

[76] Bayindir M., Aydin K., Ozbay E., Markos P., Soukoulis C.M. (2002). Transmission properties of composite metamaterials in free space. Appl. Phys. Lett..

[77] Greegor R.B., Parazzoli C.G., Li K., Tanielian M.H. (2003). Origin of dissipative losses in negative index of refraction materials. Appl. Phys. Lett..

[78] Yen T.J., Padilla W.J., Fang N., Vier D.C., Smith D.R., Pendry J.B., Basov D.N., Zhang X. (2004). Terahertz magnetic response from artificial materials. Science.

[79] Katsarakis N., Konstantinidis G., Kostopoulos A., Penciu R.S., Gundogdu T.F., Kafesaki M., Economou E.N., Koschny T., Soukoulis C.M. (2005). Magnetic response of split-ring resonators in the far-infrared frequency regime. Opt. Lett..

[80] Linden S., Enkrich C., Wegener M., Zhou J., Koschny T., Soukoulis C.M. (2004). Magnetic response of metamaterials at 100 Terahertz. Science.

[81] Zhang S., Fan W., Minhas B.K., Frauenglass A., Malloy K.J., Brueck S.R.J. (2005). Midinfrared Resonant Magnetic Nanostructures Exhibiting a Negative Permeability. Phys. Rev. Lett..

[82] Enkrich C., Wegener M., Linden S., Burger S., Zschiedrich L., Schmidt F., Zhou J.F., Koschny Th., Soukoulis C.M. (2005). Magnetic Metamaterials at Telecommunication and Visible Frequencies. Phys. Rev. Lett..

[83] Nagpal P., Lindquist N.C., Oh S.-H., Norris D.J. (2009). Ultrasmooth Patterned Metals for Plasmonics and Metamaterials. Science.

[84] Xu X., Peng B., Li D., Zhang J., Wong L.M., Zhang Q., Wang S., Xiong Q. (2011). Flexible visible-infrared metamaterials and their applications in highly sensitive chemical and biological sensing. Nano Lett..

[85] Cao C., Zhang J., Wen X., Dodson S.L., Dao N.T., Wong L.M., Wang S., Li S., Phan A.T., Xiong Q. (2013). Metamaterials-based label-free nanosensor for conformation and affinity biosensing. ACS Nano.

[86] Cao C., Zhang J., Li S., Xiong Q. (2014). Intelligent and ultrasensitive analysis of mercury trace contaminants via plasmonic metamaterial-based surface-enhanced Raman spectroscopy. Small.

[87] Shioi M., Jans H., Lodewijks K., Dorpe P.V., Lage L., Kawamura T. (2014). Tuning the interaction between propagating and localized surface plasmons for surface enhanced Raman scattering in water for biomedical and environmental applications. Appl. Phys. Lett..

[88] Guddala S., Rao D.N., Ramakrishna S.A. (2016). Resonant enhancement of Raman scattering in metamaterials with hybrid electromagnetic and plasmonic resonances. J. Opt..

[89] McFarland A.D., Young M.A., Dieringer J.A., Van Duyne R.P. (2005). Wavelength-scanned surface-enhanced Raman excitation spectroscopy. J. Phys. Chem. B.

[90] Chu Y., Banaee M.G., Crozier K.B. (2010). Double-resonance plasmon substrates for surface-enhanced raman scattering with enhancedment at excitation and stokes frequencies. ACS Nano.

[91] Jiao Y., Ryckman J.D., Koktysh D.S., Weiss S.M. (2013). Controlling surface enhanced Raman scattering using grating-type patterned nanoporous gold substrates. Opt. Mater. Express.

[92] Zhang X., Zheng Y., Liu X., Lu W., Dai J., Lei D.Y. (2015). MacFarlane, D.R. Hierarchical porous plasmonic metamaterials for reproducible ultrasensitive surface-enhanced Raman spectroscopy. Adv. Mater..

[93] Fano U. (1961). Effects of configuration interaction on intensities and phase shifts. Phys. Rev..

[94] Fan J.A., Bao K., Wu C., Bao J., Bardhan R., Halas N.J., Manoharan V.N., Shvets G., Nordlander P., Capasso F. (2010). Fano-like interference in self-assembled plasmonic quadrumer clusters. Nano Lett..

[95] Rahmani M., Luk’yanchuk B., Hong M. (2013). Fano resonance in novel plasmonic nanostructures. Laser Photonics Rev..

[96] He J., Wang J., Fan C., Liang E. (2015). Double Fano-type resonances in heptamer-hole array transmission spectra with high refractive index sensing. J. Mod. Opt..

[97] Zafar R., Salim M. (2015). Enhnaced figure of merit in Fano resonance-based plasmonic refractive index sensor. IEEE Sens. J..

[98] Miroshnichenko A.E., Kivshar Y.S. (2012). Fano resonances in all-dielectric oligomers. Nano Lett..

[99] Bao K., Mirin N.A., Nordlander P. (2010). Fano resonances in planar silber nanosphere clusters. Appl. Phys. A.

[100] Mirin N.A., Bao K., Nordlander P. (2009). Fano resonances in plasmonic nanoparticle aggregates. J. Phys. Chem. A.

[101] Zhang X., Shao M., Zeng X. (2016). High quality plasmonic sensors based on Fano resonances created through cascading double asymmetric cavities. Sensors.

[102] Zhan Y., Lei D.Y., Li X., Maier S.A. (2014). Plasmonic Fano resonances in nanohole quadrumers for ultra-sensitive refractive index sensing. Nanoscale.

[103] Zhang S., Chen L., Huang Y., Xu H. (2013). Reduced linewidth multipolar plasmon resonances in metal nanorods and related applications. Nanoscale.

[104] Butet J., Martin O.J.F. (2014). Refractive index sensing with Fano resonant plasmonic nanostructures: A symmetry based nonlinear approach. Nanoscale.

[105] Gallinet B., Martin O.J.F. (2013). Refractive index sensing with subradiant modes: A framework to reduce losses in plasmonic nanostructures. ACS Nano.

[106] Yanik A.A., Cetin A.E., Huang M., Artar A., Mousavi S.H., Khanikaev A., Connor J.H., Shvets G., Altug H. (2011). Seeing protein monolayers with naked eye through plasmonic Fano resonances. PNAS.

[107] Mukherjee S., Sobhani H., Lassiter J.B., Bardhan R., Nordlander P., Halas N.J. (2010). Fanoshells: Nanoparticles with built-in Fano resonances. Nano Lett..

[108] Sonnefraud Y., Verellen N., Sobhani H., Vandenbosch A.E., Moshchalkov V.V., Van Dorpe P., Nordlander P., Maier S.A. (2010). Experimental realization of subradiant, superradiant, and Fano resonances in ring/disk palsmonic nanocavities. ACS Nano.

[109] Semouchkina E., Duan R., Semouchkin G., Pandey R. (2015). Sensing based on Fano-type resonance response of all-dielectric metamaterials. Sensors.

[110] Hao F., Sonnefraud Y., Dorpe P.V., Maier S.A., Halas N.J., Nordlander P. (2008). Symmetry breaking in plasmonic nanocavities: Subradiant LSPR sensing and a tunable Fano resonance. Nano Lett..

[111] Christ A., Martin O.J.F., Ekinci Y., Gippius N.A., Tikhodeev S.G. (2008). Symmetry breaking in a plasmonic metamaterial at optical wavelength. Nano Lett..

[112] Chirst A., Ekinci Y., Solak H.H., Gippius N.A., Tikhodeev S.G., Martin O.J. (2007). Controlling the Fano interference in a plasmonic lattice. Phys. Rev. B.

[113] Du L.-H., Li J., Liu Q., Zhao J.-H., Zhu L.-G. (2017). High-Q Fano-like resonance based on a symmetric dimer structure and its terahertz sensing application. Opt. Express.

[114] Artar A., Yanik A.A., Altug H. (2011). Multispectral plasmon induced transparency in coupled meta-atoms. Nano Lett..

[115] Fedotov V.A., Rose M., Prosvirin S.L., Papasimakis N., Zheludev N.I. (2007). Sharp trapped-mode resonances in planar metamaterials with a broken structural symmetry. Phys. Rev. Lett..

[116] Singh R., Al-Naib I., Koch M., Zhang W. (2011). Sharp Fano resonances in THz metamaterials. Opt. Express.

[117] Cao W., Singh R., Al-Naib I., He M., Taylor A.J., Zhang W. (2012). Low-loss ultra-high-Q dark mode plasmonic Fano metamaterials. Opt. Lett..

[118] Singh R., Cao W., Al-Naib I., Cong L., Withayachumnankul W., Zhang W. (2014). Ultrasensitive terahertz sensing with high-Q Fano resonances in metasurfaces. Appl. Phys. Lett..

[119] Nikolaenko A.E., De Angelis F., Boden S.A., Papasimaks N., Ashburn P., Fabrizio E.D., Zheludev N.I. (2010). Carbon nanotubes in a photonic metamaterial. Phys. Rev. Lett..

[120] Papsimkis N., Fedotov V.A., Zheludev N.I. (2008). Metmaterial analog of electromagnetically induced transparency. Phys. Rev. Lett..

[121] Sherry L.J., Jin R., Mirkin C.A., Schatz G.C., Van Duyne R.P. (2006). Localized surface plasmon resonance spectroscopy of single silver triangular nanoprisms. Nano Lett..

[122] Moritake Y., Kanamori Y., Hane K. (2014). Experimental demonstration of sharp Fano resonance in optical metamaterials composed of asymmetric double bars. Opt. Lett..

[123] Wu C., Khanikaev A.B., Adato R., Arju N., Yanik A.A., Altug H., Shvets G. (2012). Fano-resonant asymmetric metamaterials for ultrasensitive spectroscopy and identification of molecular monolayers. Nat. Mater..

[124] Reinhard B., Schmitt K.M., Wollrab V., Neu J., Beigang R., Rahm M. (2012). Metamaterials near-field sensor for deep-subwavelength thickness measurements and sensitive refractometry in the terahertz frequency range. Appl. Phys. Lett..

[125] Mock J.J., Hill R.T., Degiron A., Zauscher S., Chilkoti A., Smith D.R. (2008). Distance-dependent plasmon resonant coupling between a gold nanoparticle and gold film. Nano Lett..

[126] Knight M.W., Wu Y., Lassiter J.B., Nordlander P., Halas N.J. (2009). Substrate matter: Influence of an adjacent dielectric on an individual plasmonic nanoparticle. Nano Lett..

[127] Zhang S., Bao K., Halas N.J., Xu H., Nordlander P. (2011). Substrate-induced Fano resonances of a plasmonic nanocube: A route to increased-sensitivity localized surface plasmon resonance revealed. Nano Lett..

[128] Shen Y., Zhou J., Liu T., Tao Y., Jiang R., Liu M., Xiao G., Zhu J., Zhou Z.-K., Wang X. (2013). Plasmonic gold mushroom arrays with refractive index sensing figures of merit approaching the theoretical limit. Nat. Commun..

[129] Amin M., Farhat M., Bağcı H. (2013). A dynamically reconfigurable Fano metamaterial through graphene tuning for switching and sensing applications. Sci. Rep..

[130] Lee K.-L., Wu S.-H., Lee C.-W., Wei P.-K. (2011). Sensitive biosensors using Fano resonance in single gold nanoslit with periodic grooves. Opt. Express.

[131] Lee K.-L., Huang J.-B., Chang J.-W., Wu S.-H., Wei P.-K. (2015). Ultrasensitive biosensors using enhanced Fano resonances in capped gold nanoslit arrays. Sci. Rep..

[132] Lee K.-L., Hsu H.-Y., You M.-L., Chang C.-C., Pan M.-Y., Shi X., Ueno K., Misawa H., Wei P.-K. (2017). Highly sensitive aluminum-based biosensors using tailorable Fano resonances in capped nanostructures. Sci. Rep..

[133] Garnett J.C.M. (1904). Colours in Metal Glasses and in Metallic Films. Proc. R. Soc. Lond.

[134] Vasilantonakis N., Nasir M.E., Dickson W., Wurtz G.A., Zayats A.V. (2015). Bulk plasmon-polaritons in hyperbolic nanorod metamaterial waveguides. Laser Photonics Rev..

[135] Kabashin A.V., Evans P., Pastkovsky S., Hendren W., Wurtz G.A., Atkinson R., Pollard R., Podolskiy V.A., Zayats A.V. (2009). Plasmonic nanorod metamaterials for biosensing. Nat. Mater..

[136] Sreekanth K.V., Alapan Y., ElKabbash M., Ilker E., Hinczewski M., Gurkan U.A., Luca A.D., Strangi G. (2016). Extreme sensitivity biosensing platform based on hyperbolic metamaterials. Nat. Mater..

[137] Sreekanth K.V., Alapan Y., ElKabbash M., Wen A.M., Ilker E., Hinczewski M., Gurkan U.A., Steinmetz N.F., Strangi G. (2016). Enhancing the Angular Sensitivity of Plasmonic Sensors Using Hyperbolic Metamaterials. Adv. Opt. Mater..

[138] Yu N., Genevet P., Kats M.A., Aieta F., Tetienne J.P., Capasso F., Gaburro Z. (2011). Light propagation with phase discontinuities: Generalized laws of reflection and refraction. Science.

[139] Aieta F., Genevet P., Yu N., Kats M.A., Gaburro Z., Capasso F. (2012). Out-of-plane reflection and refraction of light by anisotropic optical antenna metasurfaces with phase discontinuities. Nano Lett..

[140] Khorasaninejad M., Capasso F. (2015). Broadband multifunctional efficient meta-gratings based on dielectric waveguide phase shifters. Nano Lett..

[141] Aieta F., Genevet P., Kats M.A., Yu N., Blanchard R., Gaburro Z., Capasso F. (2012). Aberration-free ultrathin flat lenses and axicons at telecom wavelengths based on plasmonic metasurfaces. Nano Lett..

[142] Ni X., Ishii S., Kildishev A.V., Shalaev V.M. (2013). Ultra-thin, planar, Babinet-inverted plasmonic metalenses. Light Sci. Appl..

[143] Chen X., Huang L., Mühlenbernd H., Li G., Bai B., Tan Q., Jin G., Qiu C.-W., Zhang S., Zentgraf T. (2012). Dual-polarity plasmonic metalens for visible light. Nat. Commun..

[144] Zheng G., Mühlenbernd H., Kenney M., Li G., Zentgraf T., Zhang S. (2015). Metasurface holograms reaching 80% efficiency. Nat. Nanotechnol..

[145] Huang L., Chen X., Mühlenbernd H., Zhang H., Chen S., Bai B., Tan Q., Jin G., Cheah K.-W., Qiu C.-W. (2013). Three-dimensional optical holography using a plasmonic metasurface. Nat. Commun..

[146] Ni X., Kildishev A.V., Shalaev V.M. (2013). Metasurface holograms for visible light. Nat. Commun..

[147] Pancharatnam S. (1956). Generalized theory of interference and its applications. Proc. Indian Acad. Sci..

[148] Berry M.V. (1984). Quantal Phase Factors Accompanying Adiabatic Changes. Proc. R. Soc. Lond. Ser. A Math. Phys. Eng. Sci..

[149] Bomzon Z.E., Biener G., Kleiner V., Hasman E. (2002). Space-variant Pancharatnam–Berry phase optical elements with computer-generated subwavelength gratings. Opt. Lett..

[150] Hasman E., Bomzon Z.E., Niv A., Biener G., Kleiner V. (2002). Polarization beam-splitters and optical switches based on space-variant computer-generated subwavelength quasi-periodic structures. Opt. Commun..

[151] Treibitz T., Schechner Y.Y. (2009). Active polarization descattering. IEEE Trans. Pattern Anal. Mach. Intell..

[152] Sparks W., Hough J.H., Germer T.A., Robb F., Kolokolova L. (2012). Remote sensing of chiral signatures on Mars. Planet. Space Sci..

[153] Craig A.B. (2013). Understanding Augmented Reality: Concepts and Applications.

[154] Sparks W.B., Hough J.H., Kolokolova L., Germer T.A., Chen F., DasSarma S., DasSarma P., Robb F.T., Manset N., Reid I.N. (2009). Circular polarization in scattered light as a possible biomarker. J. Quant. Spectrosc. Radiat. Transf..

[155] Sparks W.B., Hough J., Germer T.A., Chen F., DasSarma S., DasSarma P., Robb F.T., Manset N., Kolokolova L., Reid N. (2009). Detection of circular polarization in light scattered from photosynthetic microbes. Proc. Natl. Acad. Sci. USA.

[156] Khorasaninejad M., Crozier K.B. (2014). Silicon nanofin grating as a miniature chirality-distinguishing beam-splitter. Nat. Commun..

[157] Turner M.D., Saba M., Zhang Q., Cumming B.P., Schröder-Turk G.E., Gu M. (2013). Miniature chiral beamsplitter based on gyroid photonic crystals. Nat. Photonics.

[158] Gansel J.K., Thiel M., Rill M.S., Decker M., Bade K., Saile V., Freymann G.V., Linden S., Wegener M. (2009). Gold helix photonic metamaterial as broadband circular polarizer. Science.

[159] Kaschke J., Blume L., Wu L., Thiel M., Bade K., Yang Z., Wegener M. (2015). A helical metamaterial for broadband circular polarization conversion. Adv. Opt. Mater..

[160] Kuzyk A., Schreiber R., Fan Z., Pardatscher G., Roller E.-M., Högele A., Simmel F.C., Govorov A.O., Liedl T. (2012). DNA-based self-assembly of chiral plasmonic nanostructures with tailored optical response. Nature.

[161] Esposito M., Tasco V., Todisco F., Benedetti A., Sanvitto D., Passaseo A. (2014). Three dimensional chiral metamaterial nanospirals in the visible range by vertically compensated focused ion beam induced-deposition. Adv. Opt. Mater..

[162] Singh J.H., Nair G., Ghosh A., Ghosh A. (2013). Wafer scale fabrication of porous three-dimensional plasmonic metamaterials for the visible region: Chiral and beyond. Nanoscale.

[163] Larsen G.K., He Y., Wang J., Zhao Y. (2014). Scalable fabrication of composite Ti/Ag plasmonic helices: Controlling morphology and optical activity by tailoring material properties. Adv. Opt. Mater..

[164] Esposito M., Tasco V., Todisco F., Cuscunà M., Benedetti A., Sanvitto D., Passaseo A. (2015). Triple-helical nanowires by tomographic rotatory growth for chiral photonics. Nat. Commun..

[165] Zhao Y., Belkin M.A., Alù A. (2012). Twisted optical metamaterials for planarized ultrathin broadband circular polarizers. Nat. Commun..

[166] Yun J.-G., Kim S.-J., Yun H., Lee K., Sung J., Kim J., Lee Y., Lee B. (2017). Broadband ultrathin circular polarizer at visible and near-infrared wavelengths using a non-resonant characteristic in helically stacked nano-gratings. Opt. Express.

[167] Ke Y., Liu Z., Liu Y., Zhou J., Shu W., Luo H., Wen S. (2016). Compact photonic spin filters. Appl. Phys. Lett..

[168] Wen D., Yue F., Kumar S., Ma Y., Chen M., Ren X., Kremer P.E., Gerardot B.D., Taghizadeh M.R., Buller G.S. (2015). Metasurface for characterization of the polarization state of light. Opt. Express.

[169] Pors A., Nielsen M.G., Bozhevolnyi S.I. (2015). Plasmonic metagratings for simultaneous determination of Stokes parameters. Optica.

[170] Pors A., Bozhevolnyi S.I. (2016). Waveguide metacouplers for in-plane polarimetry. Phys. Rev. Appl..

[171] Mueller J.B., Leosson K., Capasso F. (2016). Ultracompact metasurface in-line polarimeter. Optica.

[172] Zhu A.Y., Chen W.T., Khorasaninejad M., Oh J., Zaidi A., Mishra I., Devlin R.C., Capasso F. (2017). Ultra-compact visible chiral spectrometer with meta-lenses. APL Photonics.

[173] Khorasaninejad M., Chen W.T., Oh J., Capasso F. (2016). Super-dispersive off-axis meta-lenses for compact high resolution spectroscopy. Nano Lett..

[174] Ding F., Pros A., Chen Y., Zenin V.A., Bozhevolnyi S.I. (2017). Beam-size-invarient spectropolarimeters using gap-plasmon metasurfaces. ACS Photonics.

